# Replacing Needle Injection by a Novel Waterjet Technology Grants Improved Muscle Cell Delivery in Target Tissues

**DOI:** 10.1177/09636897221080943

**Published:** 2022-04-23

**Authors:** Ruizhi Geng, Jasmin Knoll, Niklas Harland, Bastian Amend, Markus D. Enderle, Walter Linzenbold, Tanja Abruzzese, Claudia Kalbe, Elisabeth Kemter, Eckhard Wolf, Martin Schenk, Arnulf Stenzl, Wilhelm K. Aicher

**Affiliations:** 1 Department of Urology, Center for Medical Research, Eberhard Karl University of Tübingen, Tübingen, Germany; 2 Department of Urology, University of Tübingen Hospital, Eberhard Karl University of Tübingen, Tübingen, Germany; 3 Erbe Elektromedizin GmbH, Tübingen, Germany; 4 Institute of Muscle Biology and Growth, Research Institute for Farm Animal Biology, Dummerstorf, Germany; 5 Department of Molecular Animal Breeding and Biotechnology, LMU Munich, Oberschleißheim, Germany; 6 Center for Innovative Medical Models, LMU Munich, Oberschleißheim, Germany; 7 Department of Surgery, University of Tübingen Hospital, Eberhard Karl University of Tübingen, Tübingen, Germany

**Keywords:** myoblast injection, waterjet technology, cell therapy, muscle regeneration, urinary incontinence, porcine model

## Abstract

Current regimen to treat patients suffering from stress urinary incontinence often seems not to yield satisfactory improvement or may come with severe side effects. To overcome these hurdles, preclinical studies and clinical feasibility studies explored the potential of cell therapies successfully and raised high hopes for better outcome. However, other studies were rather disappointing. We therefore developed a novel cell injection technology to deliver viable cells in the urethral sphincter complex by waterjet instead of using injection needles. We hypothesized that the risk of tissue injury and loss of cells could be reduced by a needle-free injection technology. Muscle-derived cells were obtained from young male piglets and characterized. Upon expansion and fluorescent labeling, cells were injected into cadaveric tissue samples by either waterjet or injection needle. In other experiments, labeled cells were injected by waterjet in the urethra of living pigs and incubated for up to 7 days of follow-up. The analyses documented that the cells injected by waterjet *in vitro* were viable and proliferated well. Upon injection in live animals, cells appeared undamaged, showed defined cellular somata with distinct nuclei, and contained intact chromosomal DNA. Most importantly, by *in vivo* waterjet injections, a significantly wider cell distribution was observed when compared with needle injections (*P* < .05, *n* ≥ 12 samples). The success rates of waterjet cell application in living animals were significantly higher (≥95%, *n* = 24) when compared with needle injections, and the injection depth of cells in the urethra could be adapted to the need by adjusting waterjet pressures. We conclude that the novel waterjet technology injects viable muscle cells in tissues at distinct and predetermined depth depending on the injection pressure employed. After waterjet injection, loss of cells by full penetration or injury of the tissue targeted was reduced significantly in comparison with our previous studies employing needle injections.

## Introduction

Urinary incontinence (UI) is a rather frequent condition. The prevalence in adult populations was reported to range from 15% to 35%^1–4^. Stress urinary incontinence (SUI), the most common form of UI, is characterized by involuntary loss of urine under mechanical stress to the lower pelvic floor, for example, by lifting, coughing, or sneezing. In men, prostate surgery is the main risk factor for SUI. In women, SUI is associated with pregnancy and vaginal delivery. SUI is caused by a functional deficiency of the urethral sphincter complex. This may stem from loss of muscle tissue, apoptosis of muscle cells, or loss of muscular enervation^5^. When diagnosed in time, physical exercise of lower pelvic floor muscles may improve sphincter function. Exercise in combination with bio-feedback and/or electrostimulation is a suggested regimen as well^6^. Activation of muscle precursor cells residing in the sphincter tissue may contribute to functional regeneration upon exercise or electrophysiological stimulation^7^. But SUI is a condition caused by severe reduction in sphincter performance due to loss of muscle cells or muscular enervation. Therefore, treatment of SUI requires other strategies^8^. For more severe cases and after failed conservative treatment, current guidelines advice for surgical approaches: Implantation of artificial sphincters and fixing the position of the urethra by artificial supports, for example, by tapes, are common regimens SUI treatment. But the median durability and biocompatibility of implants still fall short of expectations. This motivated preclinical research and clinical feasibility studies investigating the prospects of SUI cell therapy^9–12^.

The sphincter complex includes two types of muscle tissue: the lissosphincter, composed of smooth muscles, and the rhabdosphincter, composed of striated muscles. Accordingly, cell therapy of SUI in (pre)clinical studies employed two distinct strategies: strengthening the striated muscle by injection of muscle-derived cells (MDCs)^13–15^ or improving smooth muscle function as well as the vascularization and enervation by injection of mesenchymal stromal cells (MSCs), adipose tissue–derived stromal cells (ADSCs), or related cells^11,16,17^. For cell injections, minimally invasive and transurethral approaches are preferred, and cells are injected by needles under visual control using a cystoscope. But recent animal studies provided evidence that needle injections of cells in the sphincter complex by transurethral route using a cystoscope often misplaced the cells^18,19^. Deposition of cells at suboptimal sites or even loss of cells by full penetration of the needle through the delicate urethral sphincter may in part explain the contradictory situation in reports on SUI cell therapy^10^. To improve precision of cell injection in the urethral sphincter, we developed a novel waterjet (WJ) technology and used primarily ADSCs^20–22^. By this novel technology, cells ride gently in a stream of an isotonic buffer less than 200 μm wide and, if needed, enriched by bioactive molecules such as growth factors or by components facilitating attachment of cells to the injection side^20,21^. The energy of this narrow jet is sufficient to open the smallest cavities, probably less than 500 μm wide, without direct contact of the jet’s nozzle to the tissue surface. By preselection of the injection pressure, the energy of the jet can be adapted to the tissue targeted. Thus, full penetration of the urethra by the jet can be avoided^21^. Moreover, in contrast to sturdy injection needles, a WJ does not punch “wide holes” in tissues targeted, thus reducing loss of any active components by reflux and tissue damage, inflammation, or entry of urine in submucosal layers^18,19^. To discover whether regeneration of muscular tissues was potentially facilitated by WJ injection of MDCs, we had to investigate whether myoblasts derived from satellite cells of skeletal muscle tissue can be delivered and recovered with sufficient viability upon injection in tissue samples and survive WJ injection in live animals. In this study, we present evidence that injection of MDCs by WJ in the porcine urethra delivers viable cells fast, simple, and with high precision, minimal tissue damage, and convincing yield.

## Materials and Methods

### Isolation, Cultivation, and Labeling of Porcine MDCs

MDCs were isolated from male wild-type (WT; German Landrace) or transgenic (TG) piglets expressing a near-infrared fluorescent protein (iRFP720 transgene under the control of the ubiquitous active chicken beta actin promoter)^23^ about 4 to 5 days after birth and expanded as described^24–26^. In brief, WT piglets were sacrificed using carotid artery bleeding after captive-bolt pistol. TG piglets were sedated (atropine 0.05 mg/kg, intramuscularly and azaperone 4 mg/kg, intramuscularly). After initial sedation, deep anesthesia was established by phenobarbital (150 mg/kg, intramuscularly), confirmed by checking reflexes, and the piglets were sacrificed by carotid artery bleeding. Then the dermis was cleaned and sterilized. The dermis was opened by the aid of scalpel and scissors to prepare *musculus longissimus* or *musculus semitendinosus*. Pieces of the muscles were excised aseptically (approximately 15 g wet weight), washed, and transported in enriched phosphate-buffered saline (PBS) on wet ice. Then, the tissue was minced by blade and enzymatically degraded (20 min, 37°C, agitation in digestion buffer: 0.025% trypsin, 0.2% mixed collagenases I + II, 0.01% DNase I in PBS). The supernatant was filtered (100 μm nylon strainer), sedimented (800 × *g*, 4°C, 10 min), and the pellet was resuspended in Dulbecco’s Modified Eagle Medium (DMEM), mixed with digestion buffer again for two additional rounds of tissue degradation. Muscle extracts were pooled. The MDCs were enriched by Percoll step-gradient centrifugation as described (15,000 × *g*, 4°C, 9 min)^26^. The interface containing mononuclear cells was aspirated, diluted in DMEM, and washed twice by centrifugation. Purified MDCs were expanded in type I collagen–coated flasks in growth medium containing DMEM complemented with 10% fetal bovine serum (FBS), glutamine, and antibiotics as described^24–26^. At cell densities of approximately 70% of confluence, cells were harvested, counted, split in a 1:3 ratio, and expanded further up to their third or fourth passage of *in vitro* culture to generate cells for characterization and injection experiments.

To visualize injected MDCs and discriminate them from the target tissue, cells were labeled by fluorescent dyes prior to injections in cadaveric urethra samples or in live pigs. In some experiments, MDCs expressing the iRPF720 reporter were employed. Generation of TG pigs was licensed by the Bavarian State Authorities (file # ROB-55.2-2532. Vet_02-17-136). For *in vitro* experiments, MDCs were labeled by calcein-AM and ethidium homodimer following the manual (Life/dead viability/cytotoxicity kit; Thermo Fisher Scientific, Schwerte, Germany) and injected into fresh porcine cadaveric urethra samples by Williams needle (WN; Cook Medical, Bloomington, IN, USA)^18^ or by WJ (Erbe Elektromedizin GmbH, Tübingen, Germany)^20^. For *in vivo* experiments, MDCs were labeled immediately prior to injections by PKH26 following the manual (PKH26 label kit; Thermo Fisher Scientific)^27^, washed with PBS, counted, and prepared for WJ injections. In some experiments, 70% of MDCs were labeled by PKH26 and 30% by a baculovirus system expressing a recombinant enhanced green fluorescent protein (eGFP) as fusion protein to histone 2B as requested by the supplier (CellLight BacMam 2.0; Thermo Fisher Scientific). Efficacies of cell labeling were visualized by microscopy in phase contrast transmitted light versus fluorescence mode (Axiovert A1; Zeiss, Oberkochen, Germany).

### Characterization of Porcine MDCs

For analysis of transcript expression, cells were detached by aid of trypsin-EDTA, washed twice with PBS, and sedimented in 1.5 ml centrifugation tubes. Total RNA was extracted using RNeasy mini kits following the manual (Qiagen, Hilden, Germany) and DNA was removed by DNase. Yield and purity of RNA were measured by UV-spectrophotometry (Nanodrop; Implen, München, Germany). Complementary DNA (cDNA) was reverse-transcribed following the manuals (oligo-dT priming, MMLV enzyme, 42°C, 60 min, cDNA kit; Takara Bio Inc., Kusatsu, Shiga, Japan). Transcripts encoding myostatin (*MSTN*), the transcription factors myogenic factor-5 and myogenic factor-6 (*MYF5*, *MYF6*), myogenic differentiation 1 (*MYOD1*), myosin light chain 1 (*MYL1*), as well actin (*ACTA2*) and desmin (*DES*) were detected by quantitative polymerase chain reaction (qPCR) of cDNAs using swine-specific primer pairs (Table 1) and the following amplification protocol: 2 min 94°C for separation of RNA from cDNA and 35 cycles of amplification (30 s 58°C for primer annealing, 60 s 72°C for primer extension, 30 s 94°C for melting of double stands), followed by product completion for 5 min at 72°C (LightCycler 480; SybrGreen PCR amplification kit; Roche, Basel, Switzerland). PCR product amounts of the individual myogenic target genes were normalized to β2-microglobulin (*B2M*) as a housekeeping gene and in addition to an established DNA standard in each run^31,32^. Amplifications without DNA served as negative controls. Melting point analyses and agarose gel electrophoresis of the PCR products confirmed quality and sizes of the amplifications^33^.

**Table 1.** Primers Employed for PCR.

Gene	Upper primer	Lower primer	Size	Acc. number	Ref.
*ACTA2*	CGGGCAGGTCATCACCATC	CGTGTTGGCGTAGAGGTCCTT	160	NM_001164650.1	Maak and Wicke^28^
*DES*	ACACCTCAAGGATGAGATGGC	CAGGGCTTGTTTCTCGGAAG	176	NM_001001535.1	
*MYF5*	GCTGCTGAGGGAACAGGTGGA	CTGCTGTTCTTTCGGGACCAGAC	135	NM_001278775.1	Maak and Wicke^28^
*MYF6*	CGCCATCAACTACATCGAGAGGT	ATCACGAGCCCCCTGGAAT	189	NM_001244672.1	Maak and Wicke^28^
*MYL1*	CTCTCAAGATCAAGCACTGCG	GCAGACACTTGGTTTGTGTGG	198	NM_214374.2	Maak and Wicke^28^
*MYOD1*	CACTACAGCGGTGACTCAGACGCA	GACCGGGGTCGCTGGGCGCCTCGCT	145	NM_001002824.1	Maak and Wicke^28^
*MSTN*	CCCGTCAAGACTCCTACAACA	CACATCAATGCTCTGCCAA	141	NM_214435.2	Maak and Wicke^28^
*B2M*	ACGGAAAGCCAAATTACCTGAACTG	TCTGTGATGCCGGTTAGTGGTCT	261	NM_213978.1	Kalbe et al^29^
*SRY*	GACAATCATAGCTCAAACGATG	TCTCTAGAGCCACTTTTCTCC	133	NC_010462.3	Jaillard et al^30^

PCR primer pairs for specific amplification of porcine cDNA and chromosomal DNA. The column “Gene” refers to chromosomal DNA or complementary DNA; columns “upper primer” and “lower primer” refer to all primers in 5′ > 3′ orientation; column “Size” refers to PCR product lengths according to the published sequences in base pairs (bp) and confirmed by electrophoresis in agarose gels; column “Accession Number” denotes the gene bank accession numbers (www.ncbi.nlm.nih.gov); and column “Ref.” refers to a citation where applicable.

The expression of muscle-associated proteins desmin, and fast and slow myosin was investigated on cells by immunofluorescence^34^. To this end, MDCs were seeded in coated chamber slides, incubated overnight in complete medium, washed twice with cold PBS, and fixed by methanol (10 min, −24°C). Methanol was aspirated and cells were rinsed twice with PBS at ambient temperature (AT). Unspecific binding sites were saturated by blocking buffer [5% dry milk powder in 0.1% Tween-20 in PBS (T-PBS), 37°C, 30 min]. The samples were washed twice with T-PBS. Primary antibodies reactive with porcine antigens (Table 2) were dissolved in blocking buffer and incubated in a humidified chamber in the dark at 37°C for 2 h. The primary antibodies were aspirated, and the samples were washed three times with T-PBS at AT. Then fluorescent-labeled detection antibodies were added and incubated (37°C, 1 h, dark; Table 2). The samples were washed three times with T-PBS at AT, and nuclei were stained by 4′,6-diamidino-2-phenylindole (DAPI). The cells were visualized by fluorescence microscopy (LSM 510 meta; Zeiss). Samples omitting the primary antibodies served as controls.

**Table 2.** Antibodies Employed.

Antigen	Antibody	Source	Label	Dilution	Company
Desmin	IgG, pAB	Rabbit	ø	1:200	Abcam 15200
Fast myosin	Serum	Rabbit	ø	1:200	Abcam 91506
Slow myosin	IgG, mAB	Mouse	ø	1:100	Abcam 11083
Rabbit IgG	IgG, pAB F(ab′)_2_	Donkey	FITC	1:100	Jackson 711-096-152
Mouse IgG	IgG, pAB F(ab′)_2_	Donkey	Alexa Fluor 488	1:2,000	Abcam181289

Antibodies for immunocytochemistry and immunohistochemistry. Antibodies utilized as primary antibodies (lines 1–3) and for detection of primary antibodies (lines 4, 5) of cells in chamber slides or on cryosection. FITC: fluorescein isothiocyanate; pAB: polyclonal antibody; mAB: monoclonal antibody.

### Injections of Cells in Porcine Cadaveric Urethra Samples

**Figure 1.**
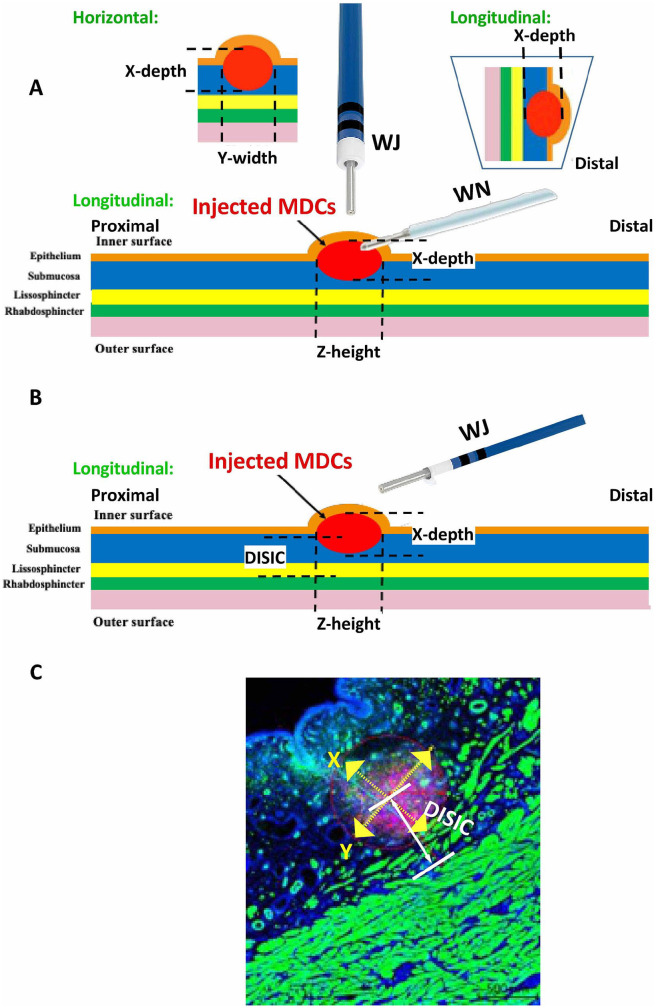
Schematic overview of cell injections. For cell injections in cadaveric urethra samples, the urethra was opened and placed on a sponge with the epithelium (ie, urothelial cell layer) facing up. (A) Injections in the submucosal layer by WN were performed at an angle of approximately 30° to 45°. For cell injections, the tip of the WN was inserted in the tissue for a few millimeters. Injections by WJ were performed vertically. The tip of the WJ lance was lowered by a gauge to the surface of the urothelial layer and moved 2 mm down without tissue penetration to avoid loss of cells by splash to the side caused by the Bernoulli effect. The X- and Y-dimensions for histologic evaluation are explained in the inserts on top. (B) For transurethral cell injections in living animals by aid of cystoscope under visual control, WJ injections were performed. After slightly tilting the device in the urethra, the flexible tip of the injection lance enabled angulated WJ injections in the urethra without penetration of the urothelium. (C) Schematic overview for determination of the distribution of cells in the tissue targeted and determination of DISIC. WN: Williams needle; WJ: waterjet; DISIC: distance between sphincter muscle and injected cells.

Fresh cadaveric female porcine urethra samples were obtained from the local abattoir. Debris was removed and a catheter was inserted in the urethra. To mimic the elasticity of lower pelvic floor tissues during cell injections, the urethra was placed on a soft sponge with the ventral side up. By scissors, the urethra was opened longitudinally to gain access to the inner side of the tube as described^20^. For needle injections, a 2-ml syringe was filled with labeled MDCs (2.4E06 ml^−1^ in complete medium) and aliquots of 250 μl were injected by WN (23 gauge; 8 mm tip; Cook Medical; Fig. 1A). For WJ injections, labeled MDCs (6.0E06 ml^−1^ in complete medium) were loaded in the dosing unit of the WJ device and aliquots of 100 μl were injected in orthogonal position using an upgraded pump and controller system (UPaCS) based on an ErbeJet2 device in an improved pressure control mode (IPCM). With this system, cell administration into the desired layer is performed by a two-phase injection. In the first phase, a high-pressure jet of a transport medium is applied at a pressure ≥60 bar to loosen the extracellular matrix of the tissue on its way to the point of treatment and to open small interconnecting micro-lacunae for the cells next to or within the muscle. In a second step, the pressure of the jet is reduced fast to a low level (eg, 10 bars) and cells are gently added to the jet exiting the nozzle^35^. In these experiments, we used pressure settings of 60 bars (Effect 60 = E60) and 80 bars (Effect 80 = E80) for tissue penetration and a pressure of 10 bar (E10) for cell injection^20^. In one set of experiments, cells injected by WN or WJ in cadaveric tissue samples were recovered by careful aspiration using a G18 needle and syringe. The injection area was flushed once with complete medium to harvest the remaining cells. Cells extracted were pooled, washed once with complete medium, seeded in coated six-well plates, and cultured in complete medium to determine the yield of fluorescent viable MDCs and to study their proliferation after *in vitro* injections as described recently^20^. In a second set of experiments, injection sides were sealed immediately after cell injections by superglue. The tissue pieces injected were excised to prepare cryosections for histology (see below).

### WJ Injection of MDCs in the Urethra of Female Pigs

The efficacy of WJ injections of MDCs was studied *in vivo* in a large animal model^18^. Healthy female landrace hybrid pigs (*n* = 24, average weight 45 kg) were purchased and adapted to the new habitat at six animals per pen in the University’s Animal Facilities under ethical husbandry and veterinarian observation for 7 days prior to surgery. On the day of surgery (day 1), animals were sedated by atropine (0.05 mg/kg, intramuscularly) and azaperone (4 mg/kg, intramuscularly) and then anesthetized (propofol, 4 mg/kg//h intravenously; fentanyl, 30–100 µg/kg/h intravenously; isoflurane, 0.8–1.6 vol%). The urethra and bladder of each animal were examined prior to the injections by cystoscopy, and healthy urine status was confirmed by urine test strips (Combur10 Test M; Roche). A sensor catheter for determination of the urethral wall pressure was introduced by aid of a cystoscope under visual control (T-Doc 7 Fr dual sensor catheter; Laborie, Enschede, The Netherlands). The urethral wall pressure and the localization of the sphincter complex were determined by urodynamic measurement (Aquarius TT UDS120; Laborie) as described^36^. Fluorescent MDCs were injected in the area of urethral wall pressure maxima (Supplemental Fig. S1) by WJ employing the UPaCS and IPCM at E60-10 and E80-10 settings (Supplemental Fig. S2). After surgical intervention, animals were either sacrificed to prepare urethral tissue samples for immediate analyses (subcutaneously, day 1) or kept in husbandry under veterinarian supervision for a follow-up of 3 or 7 days, respectively, and analyzed thereafter. The animal study was approved by the local Animal Welfare Authorities (file: CU-01/16; NTP: 33978-3-1).

### Detection of Injected Cells

For preparation of urethra samples from injected animals, pigs were sedated (atropine 0.05 mg/kg intramuscularly + azaperone 4 mg/kg intramuscularly) and then anesthetized (phenobarbital intravenously, 100 mg/kg), and reflexes were checked. Pigs were sacrificed in deep anesthesia by injection of T61 (0.3 ml/kg). Death was confirmed by checking reflexes and bladder and urethra were prepared. After retrieval, the tissue samples were transported in bags on wet ice immediately to an *In Vivo* Imaging System (IVIS Spectrum; PerkinElmer, Waltham, MA, USA). Fluorescent cells were localized in the urethra by IVIS (Supplemental Fig. S3) and the region of interest was excised. These pieces were embedded in molds in freezing compound (TissueTec O.C.T.; Sakura, Umkirch, Germany) and frozen in liquid nitrogen.

Cryosections were generated (20 μm; CM1860UV; Leica, Wetzlar, Germany), stained by DAPI, and mounted as described^21^. PKH26-labeled cells and cells expressing green fluorescent protein (GFP) were detected by fluorescence microscopy (Observer C1, LSM510 meta; Zeiss). Muscular cells and muscle tissues were visualized by incubation of cryosections with a phalloidin-iFluor488 conjugate (1:1,000; AAT Bioquest; Biomol, Hamburg, Germany). Urethral tissue samples were explored by H&E and AZAN staining as well^36^. Investigating injected MDCs in consecutive cryosections by microscopy determined the distribution of cells in the urethra depending on tissue height (Z-axis; Fig. 1). By automated lateral scanning, the width (Y-axis) and depth (X-axis) of cell distribution were determined in the area of widest distribution of fluorescent MDCs (Observer Z1 with apotome, LSM510 meta, fully automated motorized table; Zeiss). In addition, the distance between sphincter muscle and injected cells (DISIC) was determined. To measure the DISIC, the center of the injected cells was computed on stitched microscopy pictures and the mean distance of the cell center to the rhabdosphincter muscle was computed (Fig. 1C; Zen software; Zeiss). To determine whether cells were intact after WJ injections, DNA was isolated from four to six consecutive cryosections containing PKH26-positive cells. To this end, tissue was scratched off, collected in centrifugation tubes, and DNA extracted following the instructions of the kit (DNeasy blood and tissue DNA extraction kit; Qiagen). The yield and purity of chromosomal DNA were determined by UV spectroscopy (Nanodrop, Implen), and intact chromosomal DNA was confirmed by the detection of the gene encoding sex-determining region (*SRY*) using pig-specific primers (Table 1) and PCR as described above but using 60°C for primer annealing. The product quality was confirmed by melting point analysis and agarose gel electrophoresis.

### Statistics

Experimental data were recorded by proprietary software programs of the individual devices or generated by spreadsheet app (Excel; Microsoft, Albuquerque, NM, USA). For statistical analyses, two-sided unbiased *t* tests were employed (PRISM; GraphPad Software, San Diego, CA, USA). *P* values below .05 (*) or smaller were regarded significant and marked in the artwork accordingly.

## Results

### Characterization of Porcine MDCs

**Figure 2.**
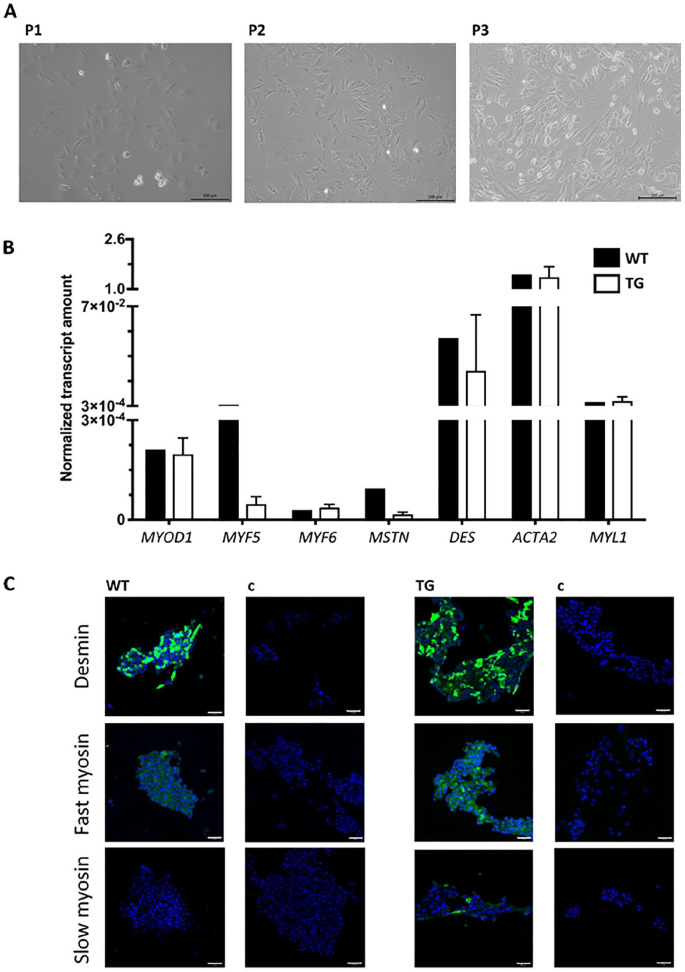
Characterization of porcine MDCs. (A) MDCs were isolated and expanded *in vitro*. The proliferating MDCs appeared as adherent populations as exemplified for the first three passages P1 to P3. Size bars = 100 μm. (B) The expression of myogenic marker transcripts was explored by RT-qPCR to compare the populations isolated from young WT and TG piglets. The graphic presents the normalized mean transcripts amounts and standard deviations (Y-axis) of seven genes as indicated (X-axis; WT: *n* = 1, TG: *n* = 4). (C) The expression of myogenic marker proteins desmin, and fast-twitch and slow-twitch myosin in WT and TG MDCs with specific primary antibodies, followed by counterstaining with FITC-labeled or Alexa 488–labeled detection antibodies (green). Nuclei were counterstained by DAPI (blue). Both populations expressed desmin. Fast myosin was expressed by WT MDCs at low and by TG MDCs at moderate levels. Slow myosin was detected only on a few cells from TG piglets. Size bars = 50 μm. Cells reacted with detection antibodies only served as controls (c). MDCs: muscle-derived cells; WT: wildtype; TG: transgenic; FITC: fluorescein isothiocyanate; DAPI: 4′,6-diamidino-2-phenylindole; RT-qPCR: reverse transcription quantitative polymerase chain reaction.

Muscle cells were isolated from muscle tissue of young male WT or TG landrace piglets and expanded as described^26^. The MDCs proliferated well *in vitro* up to the sixth passage (Fig. 2A). Prominent expression of the myogenic marker genes *MYOD1*, *DES*, *ACTA2*, and *MYL1* was confirmed by reverse transcription qPCR (RT-qPCR) in all MDC preparations in the third or fourth passage of *in vitro* culture (Fig. 2B). The expression of *MYF5* was lower in MDCs from TG piglets when compared with MDCs from WT piglets, while *MYF6* and *MSTN* were low in both populations (Fig. 2B). In addition, the expression of desmin, and fast- and slow-twitch myosin in MDCs was investigated by immune fluorescence microscopy (Fig. 2C). Desmin expression was observed in MDCs from WT and TG piglets. The expression of fast-twitch myosin was low in WT MDCs and somewhat higher in MDCs from TG animals. The expression of slow-twitch myosin was observed only in a few MDCs of TG animals (Fig. 2C).

### Injection of MDCs by WJ and WM in Tissue Samples

**Figure 3.**
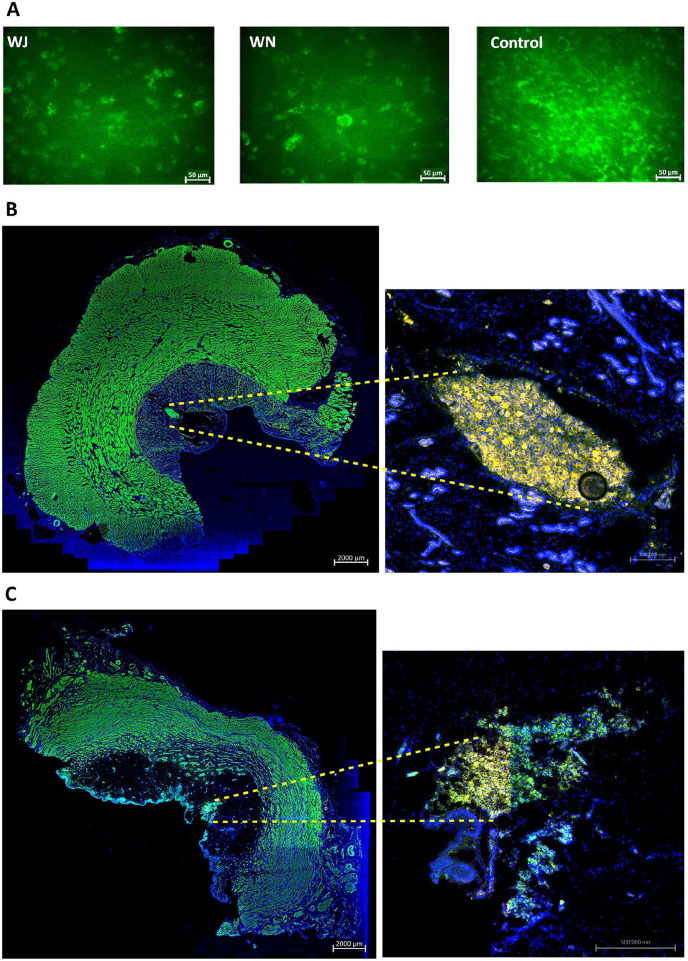
Cell injections in cadaveric tissue samples. (A) Examples of calcein-AM-labeled MDCs injected by WJ or WN in fresh porcine cadaveric urethra samples, aspirated, washed, and seeded in six-well plates for further expansion in culture. Significant differences between yield of viable cells and their proliferation rates were not observed. Noninjected calcein-AM-labeled MDCs served as controls. The pictures are representative artwork from a 5-day follow-up. Size bars indicate 50 μm. (B) TG MDCs were injected by WN in fresh porcine cadaveric urethra samples. Cryosections were generated and stained by fluorescent phalloidin to visualize the injected cells and muscular tissue. The sphincter muscle and injected MDCs appear green in the stitched overview (10× objective, left, size bar 2 mm). Injected TG MDCs appear yellow in the magnified micrograph (20× objective, right, size bar 0.2 mm). (C) TG MDCs were injected by WJ in fresh porcine cadaveric urethra samples. Cryosections were generated and stained by fluorescent phalloidin to visualize the injected cells and muscular tissue. The sphincter muscle and injected MDCs appear green in the stitched overview (10× objective, left, size bar 2 mm). Injected TG MDCs appear yellow in the magnified micrograph (20× objective, right, size bar 0.5 mm). Cell nuclei were counterstained by DAPI (blue). MDCs: muscle-derived cells; WJ: waterjet; WN: Williams needle; TG: transgenic; DAPI: 4′,6-diamidino-2-phenylindole.

To explore whether porcine MDCs can be injected by WJ with high viability employing the unmodified prototype lance, cells from male piglets were expanded, harvested, counted, and injected in capture fluid in 16 independent tests. WJ injection using this lance yielded an average normalized MDC viability of 77% ± 10%. In the next experiments, MDCs from young WT piglets were expanded and labeled by calcein-AM to flag viable cells by green fluorescence and injected by WJ or WN in fresh cadaveric urethra samples (*n* = 12 tests). In addition, injections were performed with TG MDCs (*n* = 13 tests). In one set (ie, 8× WJ injection and 4× WN injection), cells were collected immediately after injection, washed, and expanded *in vitro* for up to 5 days of culture. Prominent differences in the yields of fluorescent viable cells were not observed between samples injected by WJ versus WN. Cells directly seeded in culture vessels served as control (Fig. 3A). This corroborated that WJ injections of MDCs delivered viable cells in tissue samples at efficacies comparable to injections by WN, and at the same time confirmed recent studies employing WJ injections of porcine stromal cells^25^. In a second set of experiments, cryosections were generated from cadaveric tissue samples immediately after cell injection of TG or calcein-AM-labeled MDCs by WJ (*n* = 8) or WN (*n* = 5). Immune fluorescence microscopy determined the localization of MDCs in cadaveric tissue samples. Fluorescent cells were detected after injections by WN and WJ in all samples investigated (*n* = 13; Fig. 3B, C). Injections by WN generated fluid-filled domes in the submucosal layer of cadaveric urethrae, and cells tended to cluster at the injection front (Fig. 3B). Injections by WJ stretched the submucosal layer of cadaveric urethrae as well, but the cells tended to yield less compact clusters (Fig. 3C). Comparable patterns were observed upon WJ injection of nano- and microparticles in cadaveric samples (data not shown).

### Transurethral Injection of MDCs by WJ in Live Animals

**Figure 4.**
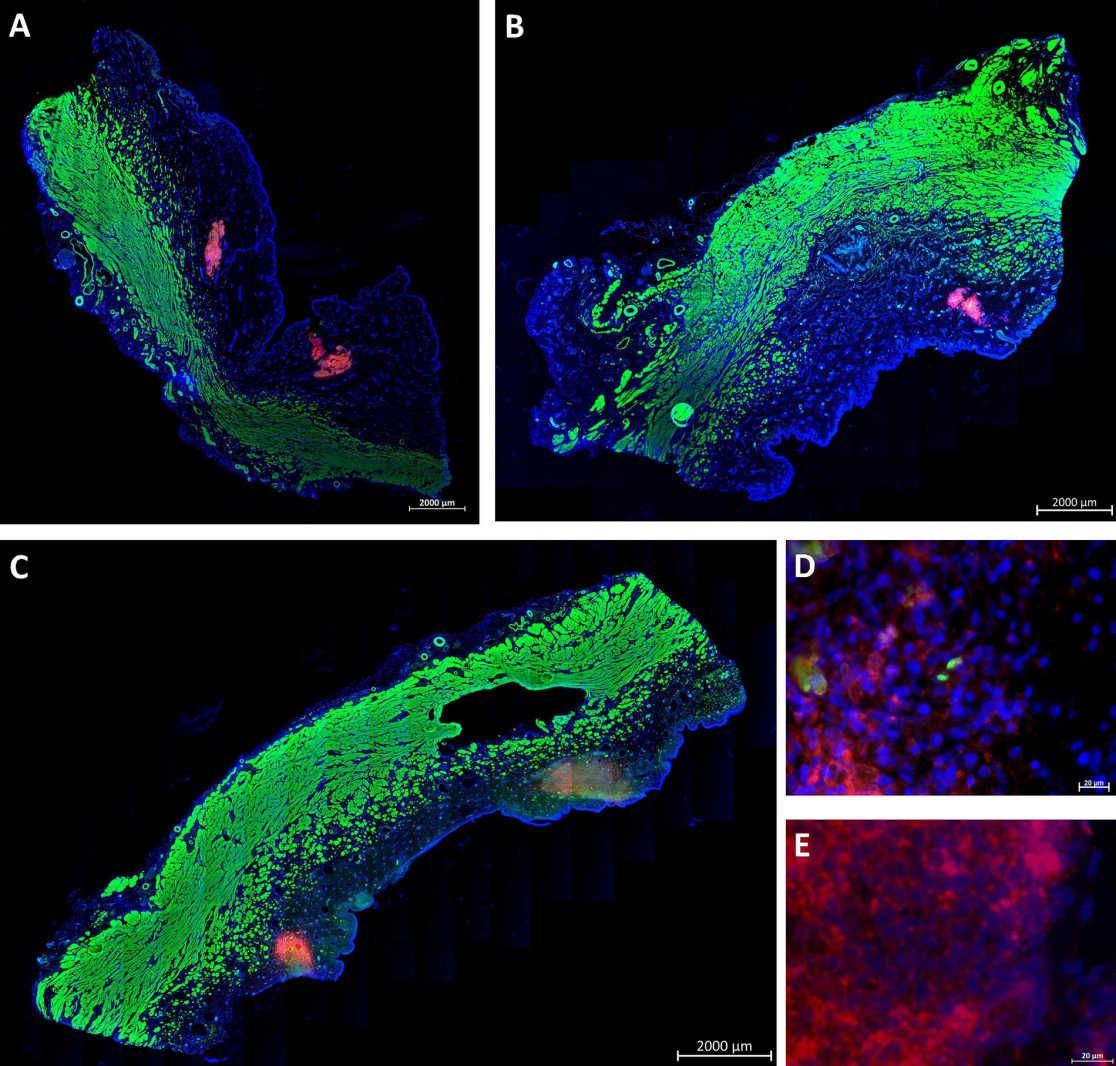
WJ injections in living animals at moderate pressure levels. Stitched micrographs (10× objective) of complete cryosection samples document PKH26-labeled fluorescent red cells in pigs treated by WJ and E60-10 protocol (A) prepared on day 1, (B) on day 3, and (C) on day 7 after injections. Cells are localized in the submucosa. Muscle tissue is stained by phalloidin and appears green, and cell nuclei are counterstained by DAPI and appear blue. Size bars = 2 mm. (D) By larger magnification (40× objective), PKH26 fluorescent cell somata surrounded by defined nuclei and detection of the expression of recombinant GFP suggest that cells injected are intact and alive after 3 days of follow-up. (E) PKH26-labeled cells were also detected after 7 days of follow-up. Size bars = 20 μm. WJ: waterjet; DAPI: 4′,6-diamidino-2-phenylindole; GFP: green fluorescent protein.

**Figure 5.**
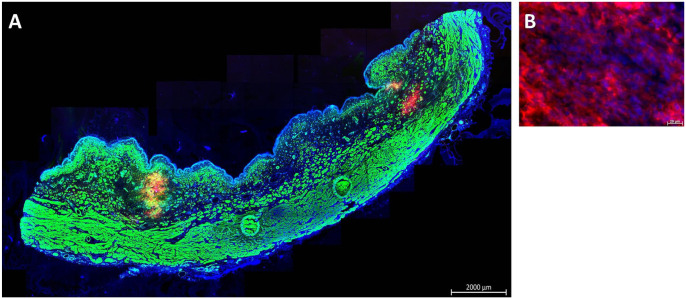
WJ injections in living animals with elevated pressure levels. Stitched micrographs (10× objective, size bar = 2 mm) of complete cryosection samples document PKH26-labeled fluorescent red cells in pigs treated by WJ and E80-10 protocol (A) on day 3 after injection. Cells are localized in the submucosa closer to the muscle. Muscle tissue is stained by phalloidin and appears green, and cell nuclei are counterstained by DAPI and appear blue. (B) By larger magnification (40× objective, size bar = 20 μm), PKH26-labeled fluorescent cell somata surrounded by defined nuclei indicate that the injected cells are intact. WJ: waterjet; DAPI: 4′,6-diamidino-2-phenylindole.

Based on the *in vitro* injections of MDCs (Fig. 3), a preclinical animal study was performed with WJ cell injections. MDCs were produced as described above, characterized, and labeled by PKH26 staining. Prior to each WJ injection, the sphincter complex was localized by measurement of the urethral wall pressure (Supplemental Fig. S1). In the first series of experiments, MDCs were injected by the improved WJ protocol (UPaCS / IPCM) at pressure settings E60-10^20^ (Supplemental Fig. S2) using a further improved prototype lance allowing sidewise injections by bending the lance nozzle. Urethral tissue samples were prepared after 1 h of *in vivo* incubation (ie, day 1) or after 3 and 7 days of follow-up. The injected cells were localized in the urethrae by IVIS (Supplemental Fig. S3), followed by (immuno)fluorescence microscopy of cryosections (Fig. 4A–C). Injected MDCs were observed in 94% of treated animals in the submucosa of the urethra (17/18 animals; Fig. 4A–C). Higher magnification presented fluorescent cell somata with intact nuclei 3 and 7 days after injection (Fig. 4D, E). In a second set of experiments, MDCs were injected by WJ using the improved UPaCS / IPCM at elevated pressure settings (E80-10) and analyzed as described above after 3 days of follow-up (Fig. 5). In 11 of 12 injection sites (ie, 91%), corresponding to treatment of six animals by E80-10 WJ injections, fluorescent MDCs were detected. In stitched micrographs, fluorescent cells were localized mainly in the submucosa of the porcine urethra (Fig. 5A). However, cells appeared somewhat closer to the lissosphincter and rhabdosphincter muscle layers when compared with E60-10 injections. Again, micrographs taken at higher magnification demonstrated fluorescent cell somata with intact cell nuclei (Fig. 5B).

**Figure 6.**
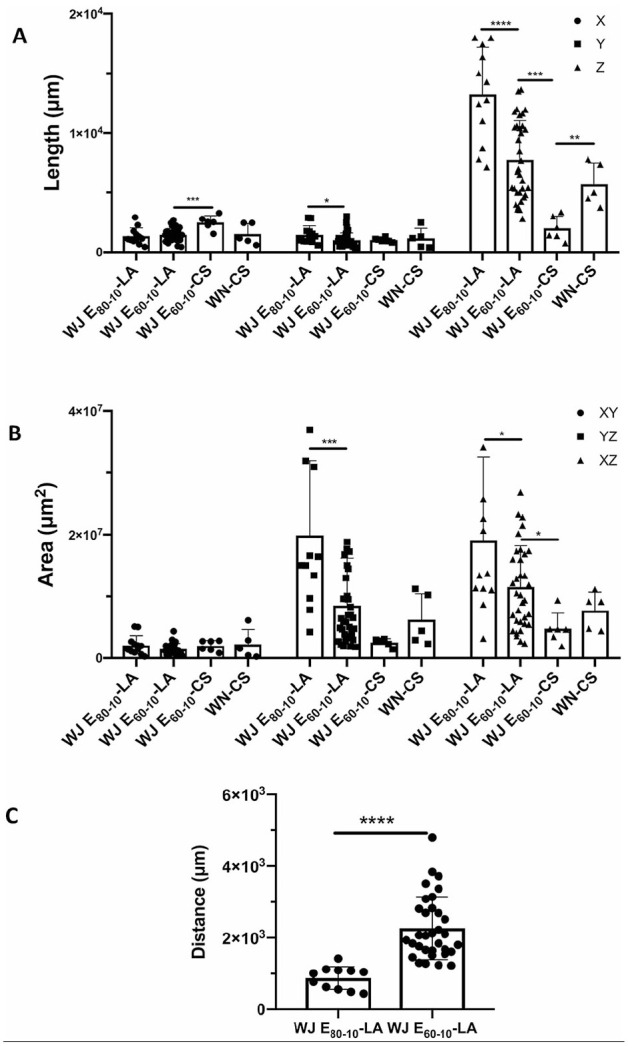
Three-dimensional cell distribution in tissues targeted. (A) Consecutive stacks of cryosections were investigated to determine the distribution of cells in three dimensions after injection by WJ using the E60-10 or E80-10 protocol in LA and after injection in CS by WJ using the E60-10 protocol or by WN as indicated. In the X- and Y-axes, only minor differences were measured. Distribution of MDCs in the height (ie, Z-axis) was significantly higher after WJ E80-10 injections in living animals when compared with WJ E60-10 injections. WJ E60-10 injections in living animals yielded a significantly higher cell distribution when compared with WJ E60-10 injections of MDCs in cadaveric tissue samples. Due to the angular WN injection in cadaveric samples, MDC distribution along the X-axis was significantly higher after WN injection in cadaver tissue samples when compared with perpendicular WJ E60-10 injections. (B) Distribution of cells was also investigated in XY-, YZ-, and XZ-planes as indicated. WJ E80-10 injections in living animals showed a significantly wider distribution in the YZ-plane when compared with WJ 60-10 injections. Comparably, in the XZ-plane, WJ E80-10 injections showed a significantly wider distribution when compared with WJ 60-10 injections. WJ E60-10 LA injections yielded significantly larger distribution in the XZ-plane when compared with injections in cadaveric urethra samples. (C) The distance between the sphincter muscle and the injected cells was significantly higher after WJ E60-10 injections in living animals when compared with WJ E80-10 injections. WJ: waterjet; LA: living animals; CS: cadaveric tissue samples; WN: Williams needle; MDCs: muscle-derived cells. Significance levels were *p ≤ 0.05, **p ≤ 0.01, ***p ≤ 0.001, ****p ≤ 0.0001.

The three-dimensional distribution of cells and the injection depths after injections by WJ versus WN in cadaveric urethra samples, as well as the distribution and injection depths of cells after WJ injection in the urethra of living cells were measured in consecutive cryosections by fluorescence microscopy (Figs. 1 and 6). Rather, narrow cell distribution and no major differences were computed in the X- and Y-dimensions of MDC injections in cadaveric samples in comparison with living animal injections using the E60-10 protocol (Fig. 6A). The computed statistically significant differences between E60-10 injections in X-dimensions in living animals versus cadaveric samples as well as the significance between E80-10 and E60-10 WJ injections in living animals in Y-dimensions were considered biologically irrelevant (Fig. 6A). In contrast, significant differences were noted in the Z-dimension between injections in living animals employing the E80-10 versus E60-10 injection protocol (*n* ≤ 12, *P* < .005; Fig. 6A). Comparably, the distribution of cells along the Z-dimension after WN injections in cadaveric samples was significantly higher when compared with WJ injections using the E60-10 protocol (*n* ≥ 5, *P* < .01; Fig. 6A).

When the cell distribution was calculated from consecutive samples in a two-dimensional manner, the X-Y-distribution (“depth–width”) did not differ between E60-10 WJ and WN injections in living animals versus cadaveric samples (Fig. 6B). But in Y-Z-dimensions (“width-height”), the E80-10 in the injection area was significantly larger when compared with E60-10 injections (*n* ≥ 12, *P* < .005; Fig. 6B), while E60-10 WJ injections in cadaveric samples in comparison with WN injections did not yield different cell distributions in the Y-Z-dimensions (Fig. 6B). Comparing the cell distribution in the X-Z-plane (“depth–height”), significant differences were obtained upon WJ injections using the E60-10 versus E80-10 mode in living animals (*n* ≥ 12, *P* < .05; Fig. 6B). WJ injections in the elevated pressure mode E80-10 generated a wider cell distribution in X-Z-dimensions.

**Figure 7.**
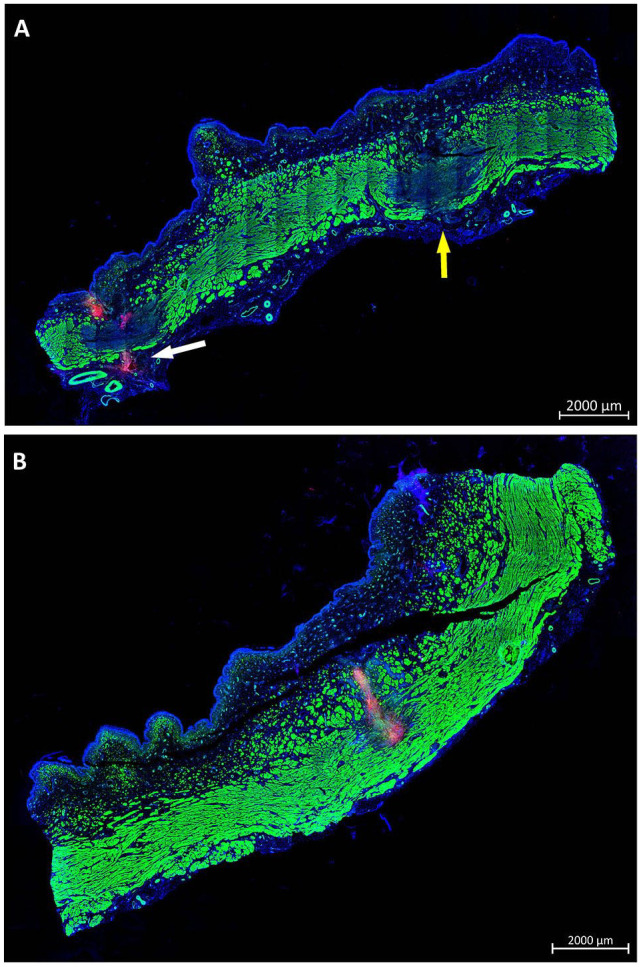
Injection depth reached by WJ in living animals. (A) Cryosamples were generated after WJ injections in living animals and stained by phalloidin and DAPI to label muscle in green and cell nuclei in blue. WJ E80-10 injection of MDCs caused full penetrations on both lateral injection spots in one of six pigs. In one penetration spot, PKH26-labeled MDCs were not detected (yellow arrow), while on the other site PKH26-labeled MDCs were observed outside the urethra (white arrow). (B) WJ E60-10 injections yielded a deep penetration in 1 of 18 pigs, and PKH26-labeled MDCs were detected in the muscular layers of the sphincter complex. Size bars = 2 mm. WJ: waterjet; MDCs: muscle-derived cells; DAPI: 4′,6-diamidino-2-phenylindole.

**Figure 8.**
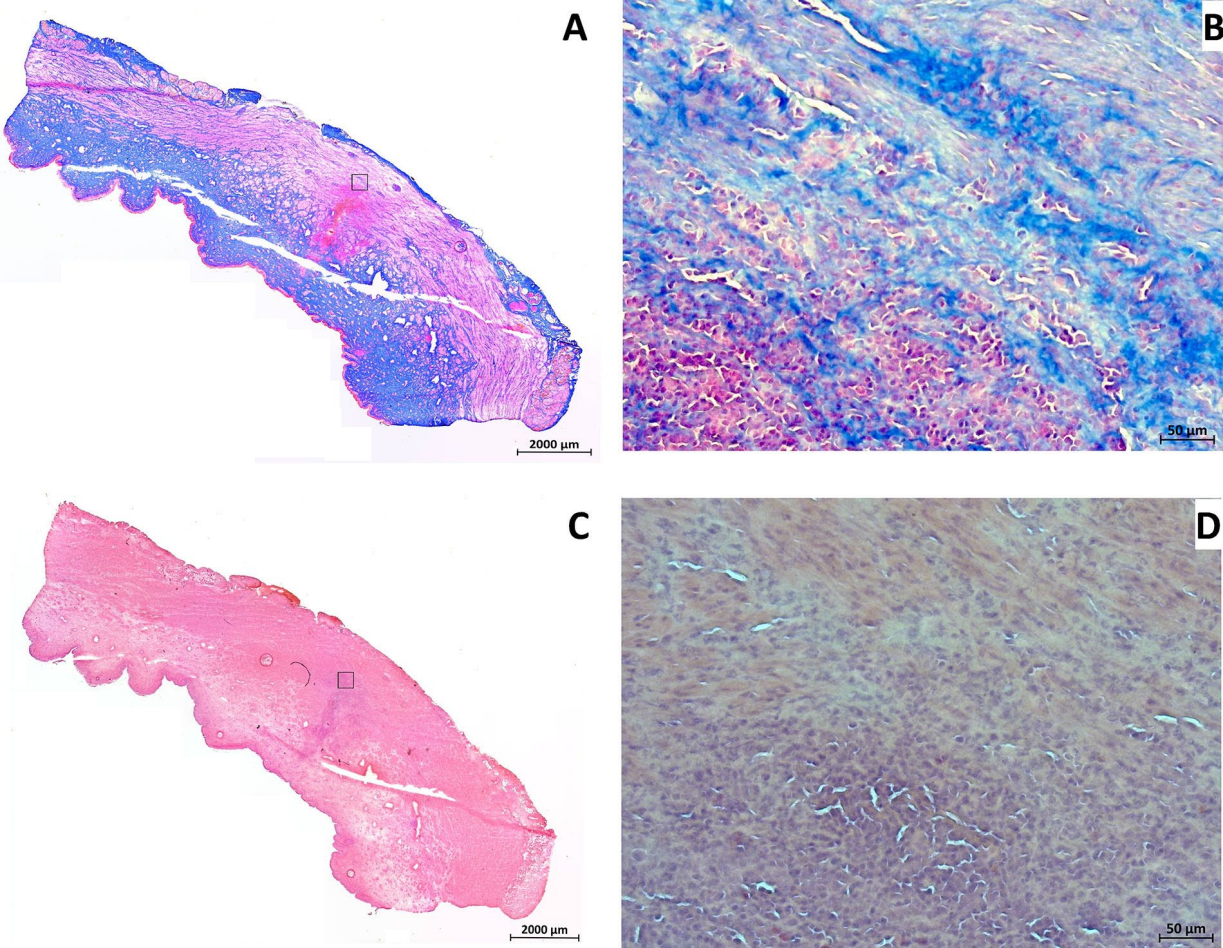
Histological analysis of the urethral tissue after WJ injection. (A) Cryosections from layers containing WJ-injected cells from animals after 7 days of follow-up were stained by AZAN to detect the injected MDCs (red color) within the submucosal connective tissue (blue color; stitched overview; 2.5× objective, size bar = 2 mm). The area of the magnified picture on the left is marked by rectangle. (B) Magnified aspect of the area of MDC injection as indicated (20× objective, size bar = 50 μm). (C) Cryosections from layers containing WJ-injected cells from animals after 7 days of follow-up were stained by H&E to visualize the tissue structure and to detect infiltration of inflammatory cells (stitched overview; 2.5× objective, size bar = 2 mm). The area of the magnified picture on the left is marked by rectangle. (D) Magnified aspect of the area of MDC injection as indicated does not show infiltration of mononuclear cells (20× objective, size bar = 50 μm). WJ: waterjet; MDCs: muscle-derived cells; H&E: hematoxylin and eosin.

In addition, the DISIC was measured after WJ injections in living animals. Using the E80-10 WJ protocol, cells were injected significantly deeper in the urethra and closer to the rhabdosphincter muscle when compared with E60-10 WJ injections (*n* ≥ 11, *P* < .005; Fig. 6C). However, a full penetration of the jet was observed in 1 of 6 animals using the E80-10 WJ protocol (83% success rate; Fig. 7A) but not in any of the 18 animals using the E60-10 protocol. In 1 of 18 animals treated by E60-10 WJ injection, MDCs were found deep in the urethra within the lissosphincter and rhabdosphicter muscles (Fig. 7B). Upon WJ injections, cells were detected by IVIS and in cryosections in samples from 23 of 24 pigs treated. The WJ injections reported here yielded an overall success rate of 95.8%. WJ injections caused a minor bleeding in 8 of 24 animals. This was observed during surgery by transurethral cystoscopy. Small injuries of the inner surface of the porcine urethra were noted in cryosection samples prepared immediately after WJ injections. No visible injuries but small hematoma was observed 3 days after WJ injection, while after 7 days of follow-up hematoma was resolved and only minor tissue coloring was noted (not shown). Infiltration of mononuclear cells was not observed in tissue samples 3 and 7 days after WJ injection (Fig. 8), and the urine status was without pathology in all animals (not shown).

### Detection of Intact Male DNA in Cryosections of Tissue Samples After WJ Injection

**Figure 9.**
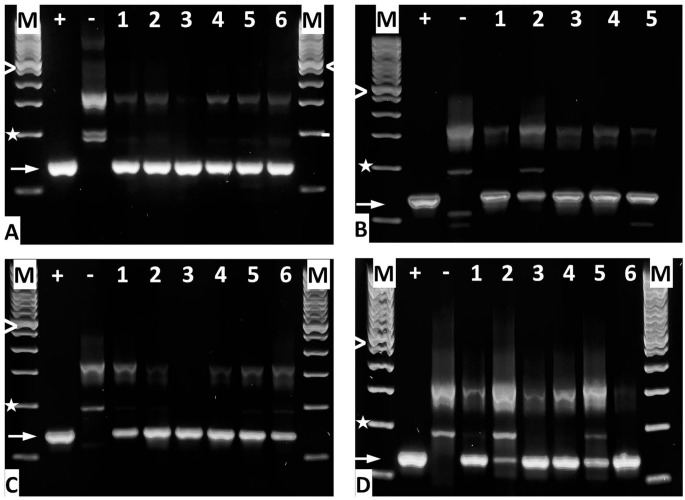
Amplification of the male-specific *SRY* gene by PCR from cryosections. DNA was prepared to detect the intact *SRY* gene by PCR after WJ of MDCs using the (A) E60-10 method at day 1 of f/u, (B) E60-10 method on day 3 of f/u, (C) E60-10 method on day 7 of f/u, and (D) E80-10 method on day 3 of f/u. “M” denotes the 100-bp DNA ladder size marker; “>” = 600 bp, “*” = 200 bp, “+” = positive control (DNA from porcine male adipocytes), “−“ = negative control (DNA from female porcine adipocytes), and “➞” = the 133-bp *SRY* PCR product. DNA samples from animals are numbered. Note that for E60-10 at 3 days of f/u, cells were found only in five of six animals treated. *SRY*: sex-determining region; WJ: waterjet; f/u: follow-up.

Injection of MDCs in cadaveric samples and retrieval for further culture indicated that WJ injections delivered viable cells at yields comparable to needle injections (Fig. 3). By fluorescence microscopy, intact nuclei of fluorescent cells indicated that the MDCs injected by WJ in living animals appeared intact (Figs. 4 and 5). Infiltration of mononuclear cells as response to necrosis of injected cells was not observed (Fig. 8). This implied that cells injected by WJ were not dead. To verify intact chromosomes in MDCs after WJ injections in living animals, DNA was isolated from consecutive cryosections containing fluorescent cells to search for the male *SRY* allele by PCR. In samples from all animals investigated, the 133-bp PCR product was detected (Fig. 9). This is evidence that after E60-10 WJ injections and follow-up of up to 7 days (in 17/18 animals injected) as well as after E80-10 WJ injections with a follow-up of 3 days (in 6/6 animals injected), sufficient numbers of male cells with intact Y-chromosomes remained in the tissue targeted (Fig. 9). From this, we infer that WJ injections delivered cells fast, precisely, and gently in the urethra in this preclinical SUI therapy model in 95.8% of animals included, without provoking a notable inflammatory response.

## Discussion

Cell therapy of SUI is not yet a standard procedure despite many successfully completed preclinical animal studies reporting promising results^9,37^. Initially, only a few clinically feasible studies reported success^4^, the quality and type of cells injected varied considerably^37^, large cohort studies with the corresponding control groups were mostly missing^38^, follow-up in many studies remained rather short, and outcome was variable and not evaluated consistently. Drop-out during clinical feasibility trials seemed not to be worth reporting. But a recent meta-analysis comparing outcome of midurethral sling surgery versus injection of minced muscular tissue versus *in vitro* expanded myoblasts suggested better efficacy of myoblast injections with a lower risk of adverse effects and less invasiveness, albeit at higher costs^39^. Improving effectiveness of SUI cell therapy was explored by complementing cell injections, for instance, by application of cytokines, chemokines, or extracellular vesicles^4,10,40^. But there is ample evidence that such factors act only for a limited time. They are rapidly adsorbed to different molecules including receptors in the region of interest; they are diluted by serum or lymph, distributed by natural movement of the tissue targeted, or degraded in time. We therefore hypothesized that effectiveness of cell therapies could also be improved further by a simplified, rapid, and even less invasive transurethral cell injection technique.

SUI cell therapies may support self-healing of the urethral sphincter muscle by activating local satellite cells^7^, by improving vascularization and reducing inflammation^41,42^, or by complementing the deficient sphincter muscle by myogenic cells^5,13–15,43,44^. But these processes may take some time. Cells injected for tissue regeneration or immune modulation can stay in situ and survive for several days^45^. This grants prolonged regenerative activity. However, after injection of cells by needle in the heart muscle, significant loss of cells in time and appearance of injected cells in remote tissues were reported^46^. We conclude that variable outcome of cell therapies and possibly even failure may in part be associated with loss of cells from the tissue targeted by reflux through the injection canal and migration of such cell by lymph or blood through the body. To reduce loss of cells, injection of regeneration competent cells in the presence of biomaterials providing domains for cell attachment was performed^47–49^. Biomaterials were designed to facilitate in situ tissue engineering as well^50^. But all these attempts included needle injections. Moreover, approval of combination therapies of cells plus bioactive components through authorities such as European Medicines Agency or Food and Drug Administration is very complex. Therefore, improving cell injection technologies in the first place without need for any other components may improve outcome of cell therapies with less efforts.

The MDCs employed in this study expressed *MYOD1*, *MYF5*, and *DES*. This suggested that the cells were enriched for proliferation-competent myogenic progenitor cells (myoblasts)^51^. The expression of *MYL1* and fast-twitch myosin implied that these cells may match the phenotype of fast-twitch muscles cells of the urethral closure complex^52^. But detailed analyses of individual subsets of the MDC phenotype of cells employed here are beyond the focus of this study. However, our study provides evidence that viable MDCs were injected in capture fluid and in cadaveric tissue samples. This confirmed our recent studies^25^. As seen before, WJ injections in cadaveric tissue tended to produce injection bubbles in the urethrae presenting as domes about 2–3 mm wide and 3–4 mm high. In cadaver samples, cells injected were not found distributed in the whole injection area but clustered in the center of the bubble. This could not be prevented by complementing the transportation fluid or injection media by gelatin, serum, or other carrier materials covering integrins and other matrix receptors to avoid cell-to-cell binding (data not shown). We consider this an artifact caused by the tissue stiffness reflecting the cells injected by WJ impulse from the tissue to the center of the bubble. Comparable patterns were observed upon injection of nano- and microparticles in cadaveric samples^20,53,54^. The significantly larger distribution of cells in the z-dimension after angulated WN injection in cadaveric tissue compared with orthogonal WJ application reflects probably efflux of MDCs through the canal punched by the angulated needle in the tissue in combination with the fluid pressure of the injection dome. The significant differences in Z-distribution between WJ injections in living animals and WJ injections in cadaveric tissue may in part be explained by differences in tissue elasticity.

We noted small bleeding during WJ injection in living animals and small hematoma right after it. But hematoma was resolved within a week’s time completely. Infiltration of mononuclear cells was not observed 1 week after injection, although pigs did not get any immune suppressive treatment such as corticosteroids, tacrolimus, or ciclosporin^55^. This confirmed that WJ did not cause major injury, and the small injection canal excised by the narrow WJ was probably spontaneously closing. Urine or infectious microorganisms were therefore not intruding the urethra to a relevant extent, thus facilitating rapid self-healing of the injection site. This self-sealing of epithelia and submucosal tissues after WJ injections is a well-described observation in gastrointestinal WJ applications and clearly marks a key advantage of this novel method^56–58^.

In our recent study, transurethral injections in the porcine urethra by WN reported frequent misplacement or loss of cells (*n* = 96 female pigs). Cells were detected in the urethral mucosa or muscle only in about 50% of animals investigated^18^. Others reported limited accuracy of cystoscope-mediated needle injections as well^19^. In contrast, upon transurethral WJ injection, fluorescent MDCs were found in urethrae in 95% of animals included (23/24) by IVIS in tissue samples and by fluorescence microscopy in the corresponding cryosections. Moreover, full penetration of the urethra, often observed after injection of cells by WN^18^, was noticed only in one pig after an E80-10 WJ injection, but not at all after E60-10 WJ injection. On the other hand, the E80-10 injection delivered cells significantly closer to the urethral muscle layer when compared with E60-10 injection, corroborating our recent study with stromal cells^21^. However, in (pre)clinical situations, the deeper penetration of the E80-10 jet and precise delivery of cells close to the sphincter muscle must be balanced with the elevated risk of cell loss by full penetration and raised up tissue injury. In this study, we also did not investigate the localization and distribution of cells after two versus four WJ injections. Due to the comparably low impact of the E60-10 WJ, repeated E60-10 injections could improve the distribution and place more cells closer to the urethral muscle when compared with two E80-10 applications without increasing the risk of unwanted side effects. Moreover, a precise, deeper, and wider delivery of cells may be achieved by adjusting the duration of the two-phase injection. Here, we held the duration constant to avoid any parameter changes during the study. But this remains to be investigated in the next level of studies. Investigation of cell survival upon needle injection reported that slow flow rates decreased the percentage of viable cells delivered and increased the percentage of cells undergoing apoptosis within 2 days^59^. This result is in favor of short contact times of cells to any narrow injection devices. The two-phased WJ technology grants such a short transportation time.

Detection of the *SRY* gene by PCR in samples from animals 3 and 7 days after WJ injection documented that the injected male cells contained sufficiently intact DNA for amplification of this allele. As this experiment was performed as end-point PCR, a quantification of the products to determine the number of intact cells is technically impossible. But it supports the notion that intact appearing cells with defined nuclei were observed immediately after WJ injection, as well as after 3 and 7 days of follow-up. Evidence for necrosis of injected cells or tissue nearby was not detected by H&E staining of cryosections. However, in the preclinical context of this study, investigation of cell viabilities is only the first level of evaluation. To examine the efficacy of the WJ technology for the clinical use intended, future experiments must include additional studies to determine optimal cell doses, single versus multiple injections in one session, repeating cell injections during follow-up, and possibly other therapies in a suitable animal model documenting functional sphincter regeneration. To this end, a porcine model of UI was developed recently^36^. But these aspects were not yet addressed in our current studies.

## Conclusion

Based on this preclinical model of cell therapy of UI, we conclude that MCDs can be injected by cystoscope under visual control precisely and close to the urethral sphincter muscle by the novel WJ technology. Using the moderate E60-10 pressure mode, cells are delivered with significantly higher success rates when compared with cell injections by WN and appear intact during a follow-up of up to 7 days. However, the regenerative potential of MDCs to regenerate a deficient sphincter muscle must be investigated in an animal model with UI in future studies.

## Supplemental Material

sj-docx-1-cll-10.1177_09636897221080943 – Supplemental material for Replacing Needle Injection by a Novel Waterjet Technology Grants Improved Muscle Cell Delivery in Target Tissues Click here for additional data file. Supplemental material, sj-docx-1-cll-10.1177_09636897221080943 for Replacing Needle Injection by a Novel Waterjet Technology Grants Improved Muscle Cell Delivery in Target Tissues by Ruizhi Geng, Jasmin Knoll, Niklas Harland, Bastian Amend, Markus D. Enderle, Walter Linzenbold, Tanja Abruzzese, Claudia Kalbe, Elisabeth Kemter, Eckhard Wolf, Martin Schenk, Arnulf Stenzl and Wilhelm K. Aicher in Cell Transplantation

sj-docx-2-cll-10.1177_09636897221080943 – Supplemental material for Replacing Needle Injection by a Novel Waterjet Technology Grants Improved Muscle Cell Delivery in Target Tissues Click here for additional data file. Supplemental material, sj-docx-2-cll-10.1177_09636897221080943 for Replacing Needle Injection by a Novel Waterjet Technology Grants Improved Muscle Cell Delivery in Target Tissues by Ruizhi Geng, Jasmin Knoll, Niklas Harland, Bastian Amend, Markus D. Enderle, Walter Linzenbold, Tanja Abruzzese, Claudia Kalbe, Elisabeth Kemter, Eckhard Wolf, Martin Schenk, Arnulf Stenzl and Wilhelm K. Aicher in Cell Transplantation
