# Supplementary material for: Replacing Needle Injection by a Novel Waterjet Technology Grants Improved Muscle Cell Delivery in Target Tissues
Source: Cell Transplant. 2022 Apr 23;31:09636897221080943. doi: 10.1177/09636897221080943 (PMC9036380; doi:10.1177/09636897221080943)
Supplement: sj-docx-2-cll-10.1177_09636897221080943 – Supplemental material for Replacing Needle Injection by a Novel Waterjet Technology Grants Improved Muscle Cell Delivery in Target Tissues [file sj-docx-2-cll-10.1177_09636897221080943.docx]

**Replacing needle injection by a novel waterjet technology**

**grants improved muscle cell delivery in target tissues**

Ruizhi Geng^1^, Jasmin Knoll^1^, Niklas Harland^2^, Bastian Amend^2^, Markus D. Enderle^3^, Walter Linzenbold^3^, Tanja Abruzzese^1^, Claudia Kalbe^4^, Elisabeth Kemter^5,6^, Eckhard Wolf^5,6^, Martin Schenk^7^, Arnulf Stenzl^2^, and Wilhelm K. Aicher^1^

^1^ Dept. of Urology, Center for Medical Research, EKU, 72072 Tübingen, Germany

^2^ Dept. of Urology, University of Tübingen Hospital, EKU, 72076 Tübingen, Germany

^3^ Erbe Elektromedizin GmbH, 72072 Tübingen, Germany

^4^ Institute of Muscle Biology and Growth, Research Institute for Farm Animal Biology (FBN), 18196 Dummerstorf, Germany

^5^ Dept. of Molecular Animal Breeding and Biotechnology, LMU Munich, 85764 Oberschleißheim, Germany

^6^ Center for Innovative Medical Models (CiMM), LMU Munich, 85746 Oberschleißheim, Germany

^7^ Dept of Surgery, University of Tübingen Hospital, EKU, 72076 Tübingen, Germany

*** Correspondence:**Wilhelm K. Aicher
Department of Urology

Center for Medical Research
University of Tuebingen Hospital
Waldhoernlestrasse 22
D 72072 Tuebingen

Germany

E-mail: aicher@uni-tuebingen.de

Tel: +49 7071 298 7020

FAX: +49 7071 292 5072

**Running head:**

Needle-free waterjet injection of cells

**Abstract**

Current regimen to treat patients suffering from stress urinary incontinence often seem not to yield satisfactory improvement or may come with severe side effects. To overcome these hurdles, preclinical studies and clinical feasibility studies explored the potential of cell therapies successfully and raised high hopes for better outcome. However, other studies were rather disappointing. We therefore developed a novel cell injection technology to deliver viable cells in the urethral sphincter complex by waterjet instead of using injection needles. We hypothesized that the risk of tissue injury and loss of cells could be reduced by a needle-free injection technology. Muscle-derived cells were obtained from young male piglets and characterized. Upon expansion and fluorescent labeling, cells were injected in either cadaveric tissue samples by waterjet or injection needle. In other experiments, labelled cells were injected by waterjet in the urethra of living pigs and incubated for up to 7 days of follow-up. The analyses documented that the cells injected by waterjet *in vitro* were viable and proliferated well. Upon injection in live animals, cells appeared undamaged, showed defined cellular somata with distinct nuclei and contained intact chromosomal DNA. Most important, by *in vivo* waterjet injections a significantly wider cell distribution was observed when compared to needle injections (p<0.05, n≥ 12 samples). The success rates of waterjet cell application in living animals were significantly higher (≥ 95%, n=24) when compared to needle injections, and the injection depth of cells in the urethra could be adapted to the need by adjusting waterjet pressures. We conclude that the novel waterjet technology injects viable muscle cells in tissues at distinct and predetermined depth depending on the injection pressure employed. After waterjet injection, loss of cells by full penetration or injury of the tissue targeted were reduced significantly in comparison to our previous studies employing needle injections.

Keywords:

myoblast injection, waterjet technology, cell therapy, muscle regeneration, urinary incontinence, porcine model

# Introduction

Urinary incontinence (UI) is a rather frequent condition. Prevalence in adult populations ranging from 15% to 35% was reported^1-4^. Stress urinary incontinence (SUI), the most common form of UI, is characterized by involuntary loss of urine under mechanical stress to the lower pelvic floor e.g., by lifting, coughing, or sneezing. In men, prostate surgery is the main risk factor to suffer from SUI. In women, SUI is associated with pregnancy and vaginal delivery. SUI is caused by a functional deficiency of the urethral sphincter complex. This may stem from loss of muscle tissue, apoptosis of muscle cells, or from loss of muscular enervation^5^. When diagnosed in time, physical exercise of lower pelvic floor muscles may improve sphincter function. Exercise in combination with bio-feedback and/or electrostimulation are suggested regimen as well^6^. Activation of muscle precursor cells residing in the sphincter tissue may contribute to functional regeneration upon exercise or electrophysiological stimulation^7^. But SUI is a condition caused by severe reduction of sphincter performance due to loss of muscle cells or muscular enervation. Therefore, treatment of SUI requires other strategies^8^. For more severe cases and after failed conservative treatment current guidelines advice for surgical approaches: Implantation of artificial sphincters as well as fixing the position of the urethra by artificial supports e.g., by tapes are common regimen for SUI treatment. But the median durability and biocompatibility of implants still fall short of expectations. This motivated pre-clinical research and clinical feasibility studies investigating the prospects of SUI cell therapy^9-12^.

The sphincter complex includes two types of muscle tissue: the lissosphincter, composed of smooth muscles and the rhabdosphincter, composed of striated muscles. Accordingly, cell therapy of SUI in (pre-)clinical studies employed two distinct strategies: strengthening the striated muscle by injection of muscle derived cells^13-15^ or improving smooth muscle function as well as the vascularization and enervation by injection of mesenchymal stromal cells (MSCs), adipose tissue derived stromal cells (ADSCs), or related cells^11,16,17^. For cell injections minimally invasive and transurethral approaches are preferred, and cells are injected by needles under visual control using a cystoscope. But recent animal studies provided evidence, that needle injections of cells in the sphincter complex by transurethral route using a cystoscope often misplaced the cells^18,19^. Deposition of cells at suboptimal sites or even loss of cells by full penetration of the needle through the delicate urethral sphincter may in part explain the contradictory situation in reports on SUI cell therapy^10^. To improve precision of cell injection in the urethral sphincter we developed a novel waterjet (WJ) technology and used primarily ADSCs^20-22^. By this novel technology, cells ride gently in a stream of an isotonic buffer less than 200 μm wide, and, if needed, enriched by bioactive molecules such as growth factors or by components facilitating attachment of cells to the injection side^20,21^. The energy of this narrow jet is sufficient to open smallest cavities, probably less than 500 μm wide, without direct contact of the jet’s nozzle to the tissue surface. By preselection of the injection pressure, the energy of the jet can be adapted to the tissue targeted. Thus full penetration of the urethra by the jet can be avoided^21^. Moreover, in contrast to sturdy injection needles, a WJ does not punch “wide holes” in tissues targeted thus reducing loss of any active components by reflux and tissue damage, inflammation, or entry of urine in submucosal layers^18,19^. To discover if regeneration of muscular tissues was potentially facilitated by WJ injection of muscle-derived cells (MDCs), we had to investigate if myoblasts derived from satellite cells of skeletal muscle tissue can be delivered and recovered with sufficient viability upon injection in tissue samples and survive WJ injection in live animals. In this study we present evidence that injection of MDCs by WJ in the porcine urethra delivers viable cells fast, simple, with high precision, minimal tissue damage, and convincing yield.

# Materials and Methods

*Isolation, Cultivation, and Labeling of Porcine Muscle-derived Cells*

Muscle-derived cells (MDCs) were isolated from male wildtype (WT; German Landrace) or from transgenic (TG) piglets expressing a near infrared fluorescent protein (iRFP720 transgene under the control of the ubiquitous active chicken beta actin promoter^23^) about 4 - 5 days after birth and expanded as described^24-26^. In brief, WT piglets were sacrificed using carotid artery bleeding after captive-bolt pistol. TG piglets were sedated (Atropin 0.05 mg/kg, i.m. and Azaperon 4 mg/kg, i.m.). After initial sedation, deep anesthesia was established by Phenobarbital (150 mg/kg, i.m.), confirmed by checking reflexes, and the piglets were sacrificed by carotid artery bleeding. Then the dermis was cleaned and sterilized. The dermis was opened by aid of scalpel and scissors to prepare the *M. longissimus* or *M. semitendinosus*. Pieces of the muscles were excised aseptically (approx. 15 g wet weight), washed and transported in enriched PBS on wet ice. Then, the tissue was minced by blade, enzymatically degraded (20 min, 37°C, agitation in digestion buffer: 0.025% trypsin, 0.2% mixed collagenases I+II, 0.01% DNase I in PBS). The supernatant was filtered (100 μm nylon strainer), sedimented (800 g, 4°C, 10 min), and the pellet was resuspended in DMEM, mixed with digestion buffer again for two additional rounds of tissue degradation. Muscle extracts were pooled. The MDCs were enriched by Percoll step-gradient centrifugation as described (15 000 *g*, 4°C, 9 min)^26^. The interface containing mononuclear cells was aspirated, diluted in DMEM, and washed twice by centrifugation. Purified MDCs were expanded in type-I collagen-coated flasks in growth medium containing DMEM complemented with 10% FBS, glutamine, and antibiotics as described^24-26^. At cell densities of approximately 70% of confluence, cells were harvested, counted, split in a 1:3 ratio, and expanded further up to their third or fourth passage of *in vitro* culture to generate cells for characterization and injection experiments.

To visualize injected MDCs and discriminate them from the target tissue, cells were labeled by fluorescent dyes prior to injections in cadaveric urethra samples or in live pigs. In some experiments, MDCs expressing the iRPF720 reporter were employed. Generation of TG pigs was licensed by the Bavarian State Authorities (file # ROB-55.2-2532.Vet_02-17-136). For *in vitro* experiments, MDCs were labeled by calcein-AM and ethidium homodimer following the manual (Life/dead viability/cytotoxicity kit, Thermo Fisher Scientific, Schwerte, Germany), and injected in fresh porcine cadaveric urethra samples by Williams needle (WN; Cook Medical, Bloomington, IN, USA)^18^ or by waterjet (WJ; Erbe Elektromedizin GmbH, Tübingen, Germany)^20^. For *in vivo* experiments MDCs were labelled immediately prior to injections by PKH26 following the manual (PKH26 label kit; Thermo Fisher Scientific)^27^, washed with PBS, counted, and prepared for WJ injections. In some experiments 70% of MDCs were labelled by PKH26 and 30% by a baculovirus system expressing a recombinant enhanced green fluorescent protein (eGFP) as fusion protein to histone 2B as requested by the supplier (CellLight, BacMam 2.0;, Thermo Fisher Scientific). Efficacies of cell labeling were visualized by microscopy in phase contrast transmitted light vs. fluorescence mode (Axiovert A1; Zeiss, Oberkochen, Germany).

*Characterization of Porcine Muscle derived Cells*

For analysis of transcript expression, cells were detached by aid of trypsin-EDTA, washed twice with PBS, and sedimented in 1.5 mL centrifugation tubes. Total RNA was extracted using RNeasy mini kits following the manual (Qiagen, Hilden, Germany) and DNA was removed by DNase. Yield and purity of RNA were measured by UV-spectrophotometry (Nanodrop; Implen, München, Germany). Complementary DNA was reverse transcribed following the manuals (oligo-dT priming, MMLV enzyme, 42°C, 60 min. cDNA kit; Takara Bio Inc., Kusatsu, Shiga, Japan). Transcripts encoding myostatin (*MSTN*), the transcription factors myogenic factor-5 and -6 (*MYF5, MYF6*), and myogenic differentiation 1 (*MYOD1*), the myosin light chain 1 (*MYL1*), as well actin (*ACTA2*) and desmin (*DES*) were detected by quantitative polymerase chain reaction (qPCR) of cDNAs using swine specific primer pairs (table 1) and the following amplification protocol: 2 min 94°C for separation of RNA from cDNA, 35 cycles of amplification (30” 58°C for primer annealing, 60” 72°C for primer extension, 30” 94°C for melting of double stands), followed by product completion for 5’ at 72°C (LightCycler 480; SybrGreen PCR amplification kit; Roche, Basel, Switzerland). PCR product amounts of the individual myogenic target genes were normalized to β2-microglobulin (*B2M*) as a housekeeping gene and in addition to an established DNA standard in each run^28,29^. Amplifications without DNA served as negative controls. Melting point analyses and agarose gel electrophoresis of the PCR products confirmed quality and sizes of the amplifications^30^.

Expression of muscle-associated proteins desmin, fast and slow myosin was investigated on cells by immunofluorescence^31^. To this end, MDCs were seeded in coated chamber slides, incubated overnight in complete medium, washed twice with cold PBS, and fixed by methanol (10 min. -24°C). Methanol was aspirated and cells were rinsed twice with PBS at ambient temperature (AT). Unspecific binding sites were saturated by blocking buffer (5% dry milk powder in 0.1% Tween-20 in PBS (T-PBS), 37°C, 30 min). The samples were washed twice with T-PBS. Primary antibodies reactive with porcine antigens (table 2) were dissolved in blocking buffer and incubated in a humidified chamber in the dark at 37°C for 2 h. The primary antibodies were aspirated, and the samples were washed three times with T-PBS at AT. Then fluorescent-labelled detection antibodies were added and incubated (37°C, 1 h, dark; table 2). The samples were washed three times with T-PBS at AT, and nuclei were stained by DAPI. The cells were visualized by fluorescence microscopy (LSM 510 meta; Zeiss). Samples omitting the primary antibodies served as controls.

*Injections of Cells in Porcine Cadaveric Urethra Samples*

Fresh cadaveric female porcine urethra samples were obtained from the local abattoir. Debris was removed and a catheter was inserted in the urethra. To mimic the elasticity of lower pelvic floor tissues during cell injections, the urethra was placed on a soft sponge with the ventral side up. By scissors the urethra was opened longitudinally to gain access to the inner side of the tube as described^20^. For needle injections, a 2 mL syringe was filed with labelled MDCs (2.4E06mL^-1^ in complete medium) and aliquots of 250 μL were injected by WN (23ga; 8 mm tip; Cook Medical; Fig. 1A). For WJ injections, labelled MDCs (6.0E06mL^-1^ in complete medium) were loaded in the dosing unit of the WJ device and aliquots of 100 μL were injected in orthogonal position using an upgraded pump and controller system (UPaCS) based on an ErbeJet2 device in an improved pressure control mode (IPCM). With this system cell administration into the desired layer is performed by a two-phase injection. In the first phase, a high-pressure jet of a transport medium is applied at a pressure ≥60 bar in order to loosen the extracellular matrix of the tissue on its way to the point of treatment and to open small interconnecting micro-lacunae for the cells next to or within the muscle. In a second step, the pressure of the jet is reduced fast to a low level (e.g. 10 bars) and cells are gently added to the jet exiting the nozzle^32^. In these experiments, we used pressure settings of 60 bars (Effect 60 = E60) and 80 bars (Effect 80 = E80) for tissue penetration and a pressure of 10 bar (E10) for cell injection^20^. In one set of experiments, cells injected by WN or WJ in cadaveric tissue samples were recovered by careful aspiration using a G18 needle and syringe. The injection area was flushed once with complete medium to harvest remaining cells. Cells extracted were pooled, washed once with complete medium, seeded in coated 6-well plates, and cultured in complete medium to determine the yield of fluorescent viable MDCs, and to study their proliferation after *in vitro* injections as described recently^20^. In a second set of experiments, injection sides were sealed immediately after cell injections by superglue. The tissue pieces injected were excised to prepare cryosections for histology (see below).

*Waterjet Injection of MDCs in the Urethra of Female Pigs*

The efficacy of WJ injections of MDCs was studied *in vivo* in a large animal model^18^. Healthy female landrace hybrid pigs (n=24, average weight 45 kg) were purchased and adapted to the new habitat at 6 animals per pen in the University’s Animal Facilities under ethical husbandry and veterinarian observation for 7 days prior to surgery. On the day of surgery (day 1), animals were sedated by Atropin (0.05 mg/kg, i.m.) and Azaperon (4 mg/kg i.m.) and then anesthetized (Propofol, 4 mg/kg//h i. v.; Fentanyl, 30-100 µg/kg/h i.v.; Isofluran, 0,8-1,6 Vol %). The urethra and bladder of each animal were examined prior to the injections by cystoscopy and healthy urine status was confirmed by a urine test strips (Combur10 Test M; Roche). A sensor catheter for determination of the urethral wall pressure was introduced by aid of a cystoscope under visual control (T-Doc 7 Fr dual sensor catheter, Laborie). The urethral wall pressure and the localization of the sphincter complex were determined by urodynamic measurement (Aquarius TT UDS120; Laborie, Enschede, The Netherlands) as described^33^. Fluorescent MDCs were injected in the area of urethral wall pressure maxima (supplement Fig. S1) by WJ employing the UPaCS and IPCM at E60-10 and E80-10 settings (suppl. Fig. S2). After surgical intervention, animals were either sacrificed to prepare urethral tissue samples for immediate analyses (s.c., day 1), or kept in husbandry under veterinarian supervision for a follow-up of 3 or 7 days, respectively, and analyzed thereafter. The animal study was approved by the local Animal Welfare Authorities (file: CU-01/16; NTP: 33978-3-1).

*Detection of Injected Cells*

For preparation of urethra samples from injected animals, pigs were sedated (Atropin 0.05 mg/kg i.m. + Azaperon 4 mg/kg i.m.), and then anaesthetized (Phenobarbital i.v., 100 mg/kg), and reflexes were checked. Pigs were sacrificed in deep anesthesia by injection of T61 (0.3 ml/kg). Death was confirmed by checking reflexes and bladder and urethra were prepared. After retrieval, the tissue samples were transported in bags on wet ice immediately to an In Vivo Imaging System (IVIS Spectrum; PerkinElmer, Waltham, MA, USA). Fluorescent cells were localized in the urethra by IVIS (suppl. Fig. S3) and the region of interest was excised. These pieces were embedded in molds in freezing compound (TissueTec O.C.T.; Sakura, Umkirch, Germany) and frozen in liquid nitrogen.

Cryosections were generated (20 μm; CM1860UV; Leica, Wetzlar, Germany), stained by DAPI and mounted as described^21^. PKH26 labelled cells and cells expressing GFP were detected by fluorescence microscopy (Observer C1, LSM510 meta; Zeiss). Muscular cells and muscle tissues were visualized by incubation of cryosections with a phalloidin-iFluor488 conjugate (1:1000; AAT Bioquest, biomol, Hamburg, Germany). Urethral tissue samples were explored by HE and AZAN staining as well^33^. Investigating injected MDCs in consecutive cryosections by microscopy determined the distribution of cells in the urethra depending on tissue height (Z-axis; Fig. 1). By automated lateral scanning, the width (Y-axis) and depth (X-axis) of cell distribution were determined in the area of widest distribution of fluorescent MDCs (Observer Z1 with apotome, LSM510 meta, fully automated motorized table, Zeiss). In addition, the distance between sphincter muscle and injected cells (DISIC) was determined. To measure the DISIC, the center of the injected cells was computed on stitched microscopy pictures and the mean distance of the cell center to the rhabdosphincter muscle was computed (Fig. 1C; Zen software, Zeiss). To determine if cells were intact after WJ injections, DNA was isolated from 4 - 6 consecutive cryosections containing PKH26 positive cells. To this end, tissue was scratched off, collected in centrifugation tubes and DNA was extracted following the instructions of the kit (DNeasy blood and tissue DNA extraction kit; Qiagen). The yield and purity of chromosomal DNA were determined by UV spectroscopy (Nanodrop, Implen), and intact chromosomal DNA was confirmed by detection of the gene encoding sex determining region (*SRY*) using pig-specific primers (table 1) and PCR as described above but using 60°C for primer annealing. The product quality was confirmed by melting point analysis and agarose gel electrophoresis.

*Statistics*

Experimental data were recorded by proprietary software programs of the individual devices or generated by spreadsheet app (Excel; Microsoft, Albuquerque, NM, USA). For statistical analyses two-sides unbiased T-Tests were employed (PRISM; Graphpad Software, San Diego, CA, USA ). P values below 0.05 (*) or smaller were regarded significant and marked in the artwork accordingly.

# Results

## *Characterization of Porcine Muscle-derived Cells*

Muscle cells were isolated from muscle tissue of young male WT or TG landrace piglets and expanded as described^26^. The MDCs proliferated well *in vitro* up to the 6^th^ passage (Fig. 2A). Prominent expression of the myogenic marker genes *MYOD1*, *DES*, *ACTA2*, and *MYL1* was confirmed by RT-qPCR in all MDC preparations in the third or fourth passage of *in vitro* culture (Fig. 2B). Expression of *MYF5* was lower in MDCs from TG piglets when compared to MDCs from WT piglets, while *MYF6* and *MSTN* were low in both populations (Fig. 2B). In addition, expression of desmin, fast and slow twitch myosin in MDCs was investigated by immune fluorescence microscopy (Fig. 2C). Desmin expression was observed in MDCs from WT and TG piglets. Expression of fast twitch myosin was low in WT MDCs and somewhat higher in MDCs from TG animals. Expression of slow twitch myosin was observed only in a few MDCs of TG animals (Fig. 2C).

## *Injection of Muscle-derived Cells by Waterjet and Williams Needle in Tissue Samples*

To explore if porcine MDCs can be injected by WJ with high viability employing the unmodified prototype lance, cells from male piglets were expanded, harvested, counted, and injected in capture fluid in 16 independent tests. WJ injection using this lance yielded an average normalized MDC viability of 77±10%. In the next experiments, MDCs from young WT piglets were expanded and labeled by calcein-AM to flag viable cells by green fluorescence and injected by WJ or WN in fresh cadaveric urethra samples (n = 12 tests). In addition, injections were performed with TG MDCs (n = 13 tests). In one set (i.e., 8xWJ injection and 4x WN injections), cells were collected immediately after injection, washed, and expanded *in vitro* for up to 5 days of culture. Prominent differences in the yields of fluorescent viable cells were not observed between samples injected by WJ vs. WN. Cells directly seeded in culture vessels served as control (Fig. 3A). This corroborated that WJ injections of MDCs delivered viable cells in tissue samples at efficacies comparable to injections by WN, and at the same time confirmed recent studies employing WJ injections of porcine stromal cells^25^. In a second set of experiments, cryosections were generated from cadaveric tissue samples immediately after cell injection of TG or calcein-AM-labeled MDCs by WJ (n = 8) or WN (n = 5). Immune fluorescence microscopy determined the localization of MDCs in cadaveric tissue samples. Fluorescent cells were detected after injections by WN and WJ injections in all samples investigated (n = 13; Figs. 3B, 3C). Injections by WN generated fluid-filled domes in the submucosal layer of cadaveric urethrae and cells tended to cluster at the injection front (Fig. 3B). Injections by WJ stretched the submucosal layer of cadaveric urethrae as well, but the cells tended to yield less compact clusters (Fig. 3C). Comparable patterns were observed upon WJ injection of nano- and microparticles in cadaveric samples (data not shown).

## *Transurethral Injection of Muscle-derived Cells by Waterjet in Live Animals*

Based on the *in vitro* injections of MDCs (Fig. 3), a pre-clinical animal study was performed with WJ cell injections. MDCs were produced as described above, characterized, and labelled by PKH26 staining. Prior to each WJ injection, the sphincter complex was localized by measurement of the urethral wall pressure (Fig. S1). In the first series of experiments, MDCs were injected by the improved WJ protocol (UPaCS / IPCM) at pressure settings E60-10^20^ (Fig. S2) using a further improved prototype lance allowing sidewise injections by bending the lances nozzle. Urethral tissue samples were prepared after 1 h of *in vivo* incubation (i.e., day 1), or after 3 and 7 days of follow-up. The injected cells were localized in the urethrae by IVIS (Fig. S3), followed by (immuno-)fluorescence microscopy of cryosections (Fig. 4A-C). Injected MDCs were observed in 94% of treated animals in the submucosa of the urethra (17/18 animals; Fig. 4A-C). Higher magnification presented fluorescent cell somata with intact nuclei 3 and 7 days after injection (Fig. 4D, 4E). In a second set of experiments, MDCs were injected by WJ using the improved UPaCS / IPCM at elevated pressure settings (E80-10) and analyzed as described above after 3 days of follow-up (Fig. 5). In 11/12 injection sites (i.e., 91%), corresponding to treatment of 6 animals by E80-10 WJ injections, fluorescent MDCs were detected. In stitched micrographs, fluorescent cells were localized mainly in the submucosa of the porcine urethra (Fig. 5A). However, cells appeared somewhat closer to the lissosphincter and rhabdosphincter muscle layers when compared to E60-10 injections. Again, micrographs taken at higher magnification demonstrated fluorescent cell somata with intact cell nuclei (Fig. 5B).

The three-dimensional distribution of the cells as well as the injection depths after injections by WJ vs. WN in cadaveric urethra samples, as well as the distribution and injection depths of cells after WJ injection in the urethra of living cells were measured in consecutive cryosections by fluorescence microscopy (Figs. 1, 6). Rather narrow cell distribution and no major differences were computed in the X- and Y-dimensions of MDC injections in cadaveric samples in comparison to living animal injections using the E60-10 protocol (Fig. 6A). The computed statistically significant differences between E60-10 injections in X-dimensions in living animals vs. cadaveric samples as well as the significance between E80-10 vs. E60-10 WJ injections in living animals in the Y-dimensions were considered biologically irrelevant (Fig. 6A). In contrast, significant differences were noted in the Z-dimension between injections in living animals employing the E80-10 vs. E60-10 injection protocol (n≤12, p<0.005; Fig. 6A). Comparably, the distribution of cells along the Z-dimension after WN injections in cadaveric samples was significantly higher when compared to WJ injections using the E60-10 protocol (n≥5, p<0.01; Fig.6A).

When the cell distribution was calculated from consecutive samples in a two-dimensional manner, the X-Y-distribution (“depth-width”) did not differ between E60-10 WJ and WN injections in living animals vs. cadaveric samples (Fig. 6B). But in Y-Z-dimensions (“width-height”) the E80-10 the injection area was significantly larger when compared to E60-10 injections (n≥12, p<0.005; Fig. 6B), while E60-10 WJ injections in cadaveric samples in comparison to WN injections did not yield different cell distributions in the Y-Z-dimensions (Fig. 6B). Comparing the cell distribution in the X-Z-plane (“depth-height”), significant differences were obtained upon WJ injections using the E60-10 vs. E80-10 mode in living animals (n≥12, p<0.05. Fig. 6B). WJ injections in the elevated pressure mode E80-10 generated a wider cell distribution in X-Z-dimensions.

In addition, the distance between sphincter muscle and the injected cells (DISIC) was measured after WJ injections in living animals. Using the E80-10 WJ protocol, cells were injected significantly deeper in the urethra and closer to the rhabdosphincter muscle when compared to E60-10 WJ injections (n≥11, p<0.005, Fig. 6C). However, a full penetration of the jet was observed in 1/6 animals using the E80-10 WJ protocol (83% success rate; Fig. 7A) but not in any of the 18 animals using the E60-10 protocol. In 1/18 animals treated by E60-10 WJ injection, MDCs were found deep in the urethra within the lissosphincter and rhabdosphicter muscles (Fig. 7B). Upon WJ injections, cells were detected by IVIS and in cryosections in samples from 23/24 pigs treated. The WJ injections reported here yielded an overall success rate of 95.8%. WJ injections caused a minor bleeding in 8/24 animals. This was observed during surgery by transurethral cystoscopy. Small injuries of the inner surface of the porcine urethra were noted in cryosection samples prepared immediately after WJ injections. No visible injuries but small hematoma was observed 3 days after WJ injection, while after 7 days of follow-up hematoma was resolved and only minor tissue coloring was noted (not shown). Infiltration of mononuclear cells was not observed in tissue samples 3 and 7 days after WJ injection (Fig. 8), and the urine status was w/o pathology in all animals (not shown).

*Detection of Intact Male DNA in Cryosections of Tissue Samples after WJ Injection*

Injection of MDCs in cadaveric samples and retrieval for further culture indicated that WJ injections delivered viable cells at yields comparable to needle injections (Fig. 3). By fluorescence microscopy intact nuclei of fluorescent cells indicated that the MDCs injected by WJ in living animals appeared intact (Figs. 4, 5). Infiltration of mononuclear cells as response to necrosis of injected cells was not observed (Fig. 8). This implied that cells injected by WJ were not dead. To verify intact chromosomes in MDCs after WJ injections in living animals, DNA was isolated from consecutive cryosections containing fluorescent cells to search for the male *SRY* allele by PCR. In samples from all animals investigated the 133 bp PCR product was detected (Fig. 9). This is evidence that after E60-10 WJ injections and follow-up of up to 7 days (in 17/18 animals injected) as well as after E80-10 WJ injections with a follow-up of 3 days (in 6/6 animals injected), sufficient numbers of male cells with intact Y-chromosomes remained in the tissue targeted (Fig. 9). From this we infer that WJ injections delivered cells fast, precisely, and gently in the urethra in this preclinical SUI therapy model in 95.8 % of animals included, without provoking a notable inflammatory response.

**Discussion**

Cell therapy of stress urinary incontinence is not yet a standard procedure despite of many successfully completed pre-clinical animal studies reporting promising results^9,34^. Initially, only a few clinical feasibility studies reported success^4^, the quality and type of cells injected varied considerably^34^, large cohort studies with the corresponding control groups were mostly missing^35^, follow-up in many studies remained rather short, outcome was variable and not evaluated consistently. Drop-out during clinical feasibility trials seemed not to be worth reporting. But a recent meta-analysis comparing outcome of midurethral sling surgery vs. injection of minced muscular tissue vs. *in vitro* expanded myoblasts suggested better efficacy of myoblast injections with a lower risk of adverse effects and less invasiveness, albeit at higher costs^36^. Improving effectiveness of SUI cell therapy was explored by complementing cell injections for instance by application of cytokines, chemokines, or extracellular vesicles^4,10,37^. But there is ample evidence that such factors act only for a limited time. They are rapidly adsorbed to different molecules including receptors in the region of interest, they are diluted by serum or lymph, distributed by natural movement of the tissue targeted, or degraded in time. We therefore hypothesized that effectiveness of cell therapies could also be improved further by a simplified, rapid and even less invasive transurethral cell injection technique.

SUI cell therapies may support self-healing of the urethral sphincter muscle by activating local satellite cells^7^, by improving vascularization and reducing inflammation^38,39^ or by complementing the deficient sphincter muscle by myogenic cells^5,13-15,40,41^. But these processes may take some time. Cells injected for tissue regeneration or immune modulation can stay *in situ* and survive for several days^42^. This grants prolonged regenerative activity. However, after injection of cells by needle in the heart muscle, significant loss of cells in time and appearance of injected cells in remote tissues were reported^43^. We conclude that variable outcome of cell therapies and possibly even failure may in part be associated with loss of cells from the tissue targeted by reflux through the injection canal and migration of such cell by lymph or blood through the body. To reduce loss of cells, injection of regeneration competent cells in presence of biomaterials providing domains for cell attachment were performed^44-46^. Biomaterials were designed to facilitate *in situ* tissue engineering as well^47^. But all these attempts included needle injections. Moreover, approval of combination therapies of cells plus bioactive components through the Authorities such as EMA or FDA are very complex. Therefore, improving cell injection technologies in the first place without need for any other components may improve outcome of cell therapies with less efforts.

The MDCs employed in this study expressed *MYOD1*, *MYF5*, and *DES*. This suggested that the cells were enriched for proliferation competent myogenic progenitor cells (myoblasts)^48^. Expression of *MYL1* and fast-twitch myosin implied that these cells may match the phenotype of fast-twitch muscles cells of the urethral closure complex^49^. But detailed analyses of individual subsets of the MDC phenotype of cells employed here are beyond the focus of this study. However, our study provides evidence that viable MDCs were injected in capture fluid and in cadaveric tissue samples. This confirmed our recent studies^25^. As seen before, WJ injections in cadaveric tissue tended to produce injection bubbles in the urethrae presenting as domes about 2 – 3 mm wide and 3 – 4 mm high. In cadaver samples, cells injected were not found distributed in the whole injection area but clustered in the center of the bubble. This could not be prevented by complementing the transportation fluid or injection media by gelatin, serum, or other carrier materials covering integrins and other matrix receptors to avoid cell to cell binding (data not shown). We consider this an artifact caused by the tissue stiffness reflecting the cells injected by WJ impulse from the tissue to the center of the bubble. Comparable patterns were observed upon injection of nano- and microparticles in cadaveric samples^20,50,51^. The significantly larger distribution of cells in the z dimension after angulated WN injection in cadaveric tissue compared to orthogonal WJ application reflects probably efflux of MDCs through the canal punched by the angulated needle in the tissue in combination with the fluid pressure of the injection dome. The significant differences in Z-distribution between WJ injections in living animals vs. WJ injections in cadaveric tissue may be in part explained by differences in tissue elasticity.

We noted small bleeding during WJ injection in living animals and small hematoma right after it. But hematoma was resolved within a week’s time completely. Infiltration of mononuclear cells was not observed one week after injection, although pigs did not get any immune suppressive treatment such as corticosteroids, tacrolimus, or ciclosporin^52^. This confirmed that WJ did not cause major injury and the small injection canal excised by the narrow waterjet was probably spontaneously closing. Urine or infectious microorganisms were therefore not intruding the urethra to a relevant extent, thus facilitating rapid self-healing of the injection site. This self-sealing of epithelia and submucosal tissues after WJ injections is a well-described observation in gastrointestinal WJ applications and clearly marks a key advantage of this novel method^53-55^.

In our recent study, transurethral injections in the porcine urethra by WN reported frequent misplacement or loss of cells (n = 96 female pigs). Cells were detected in the urethral mucosa or muscle only in about 50% of animals investigated^18^. Others reported limited accuracy of cystoscope-mediated needle injections as well^19^. In contrast, upon transurethral WJ injection fluorescent MDCs were found in urethrae in 95% of animals included (23/24) by IVIS in tissue samples and by fluorescence microscopy in the corresponding cryosections. Moreover, full penetration of the urethra, often observed after injection of cells by WN^18^, was noticed only in one pig after an E80-10 WJ injection, but not at all after E60-10 WJ injection. On the other hand, the E80-10 injection delivered cells significantly closer to the urethral muscle layer when compared to E60-10 injection, corroborating our recent study with stromal cells^21^. However, in (pre-)clinical situations, the deeper penetration of the E80-10 jet and precise delivery of cells close to the sphincter muscle must be balanced with the elevated risk of cell loss by full penetration and raised up tissue injury. In this study we also did not investigate the localization and distribution of cells after two vs e.g., four WJ injections. Due to the comparably low impact of the E60-10 WJ, repeated E60-10 injections could improve the distribution and place more cells closer to the urethral muscle when compared to two E80-10 applications without increasing the risk of unwanted side effects. Moreover, a precise, deeper, and wider delivery of cells may be achieved by adjusting the duration of the two-phase injection. Here, we held the duration constant in order to avoid any parameter changes during the study. But this remains to be investigated in the next level of studies. Investigation of cell survival upon needle injection reported that slow flow rates decreased the percentage of viable cells delivered and increased the percentage of cells undergoing apoptosis within 2 days^56^. This result is in favor of short contact times of cells to any narrow injection devices. The two-phased WJ technology grants such a short transportation time.

Detection of the *SRY* gene by PCR in samples from animals 3 and 7 days after WJ injection documented that the injected male cells contained sufficiently intact DNA for amplification of this allele. As this experiment was performed as end-point PCR, a quantification of the products in order to determine the number of intact cells is technically impossible. But it supports the notion that intact appearing cells with defined nuclei were observed immediately after WJ injection, as well as after 3 and 7 days of follow-up. Evidence for necrosis of injected cells or tissue nearby were not detected by HE staining of cryosections. However, in the pre-clinical context of this study, investigation of cell viabilities is only the first level of evaluation. To examine the efficacy of the WJ technology for the clinical use intended, future experiments must include additional studies to determine optimal cell doses, single versus multiple injections in one session, repeating cell injections during follow-up, and possibly other therapies in a suitable animal model documenting functional sphincter regeneration. To this end, a porcine model of urinary incontinence was developed recently^33^. But these aspects were not yet addressed in our current studies.

**Conclusion**

Based on this pre-clinical model of cell therapy of urinary incontinence we conclude that muscle-derived cells can be injected by cystoscope under visual control precisely and close to the urethral sphincter muscle by the novel waterjet technology. Using the moderate E60-10 pressure mode cells are delivered with significantly higher success rates when compared to cell injections by Williams needle and appear intact during a follow-up of up to 7 days. However, the regenerative potential of MDCs to regenerate a deficient sphincter muscle must be investigated in an animal model with urinary incontinence in future studies.

# Author Contributions

RG, JK, NH, TA, WKA: cell production, quality management, animal study, evaluation of data. WL, MDE, development of waterjet. JK, TA, WKA: cell transduction. CK: WT cells. EK, EW: CAG-iRFP720 tg pigs. M.S.: animal husbandry. BA, MDE, AS, WKA: study design, funds. All authors contributed to the preparation of the manuscript and critically revising it.

# Ethical Approval

# This article does not contain any studies with human subjects. Ethical approval is therefore not applicable.

# Statement on Human and Animal Rights, Approval of Animal Study

This article does not contain any studies with human subjects. The animal study was approved by the State of Baden-Württemberg Animal Welfare Authorities under file number CU-01/16 and was registered in the Federal NTP Registry under file number 33978-3-1. All experiments involving animals were conducted in accordance with the guidelines and recommendations of the Federation of European Laboratory Animal Science Associations (FELASA) according to the “ARRIVE” rules and National Law^57^.

**Statement of Informed Consent**

There are no human subjects in this article. Informed consent is not applicable.

# Conflict of Interest

WL and MDE are employed by ERBE Elektromedizin GmbH. ERBE Elektromedizin GmbH and WKA have received joined public funding from the Federal Government under file # 13GW007. All other authors declare that the research presented in this study was conducted in the absence of any commercial or financial relationships that could be construed as a potential conflict of interest.

# Funding

This project was supported in part by grants from the EU to WKA (MUS.I.C., #731377); the BMBF to MDE and WKA (Multimorb-INKO, # 13GW007); the DFG to BA, AS and WKA (PoTuS, #429049495); from the EU's Horizon 2020 program to EK and EW (iNanoBIT, # 760986); from the BMBF to the German Centre for Diabetes Research to EW (DZD e.V., # 82DZD00802); Zukunftsfonds, FBN Dummerstorf to CK; and in part by institutional funds.

# Acknowledgments

The authors thank Dr. Barbara Keßler for essential logistics and tissue samples, Christine Fahrner, Andreas Fech, A. Luise Jäger, Anne Berndt, Marie Jugert-Lund, and Vera Rothfuß for experimental support, and Dr. Nicolas Beziere for IVIS analyses.

ORCID ID WKA: https://orcid.org/0000-0003-2210-6773

# References

1. Broome BAS. The impact of urinary incontinence on self-efficacy and quality of life. Health and Quality of Life Outcomes. 2003;1(1):35.

2. Irwin DE, Kopp ZS, Agatep B, Milsom I, Abrams P. Worldwide prevalence estimates of lower urinary tract symptoms, overactive bladder, urinary incontinence and bladder outlet obstruction. BJU Int. 2011;108(7):1132-8.

3. Norton P, Brubaker L. Urinary incontinence in women. Lancet. 2006;367(9504):57-67.

4. Hillary CJ, Roman S, MacNeil S, Aicher WK, Stenzl A, Chapple CR. Regenerative medicine and injection therapies in stress urinary incontinence. Nat Rev Urol. 2020;17(3):151-161.

5. Schmid FA, Williams JK, Kessler TM, Stenzl A, Aicher WK, Andersson KE, Eberli D. Treatment of Stress Urinary Incontinence with Muscle Stem Cells and Stem Cell Components: Chances, Challenges and Future Prospects. Int J Mol Sci. 2021;22(8).

6. Lukacz ES, Santiago-Lastra Y, Albo ME, Brubaker L. Urinary Incontinence in Women: A Review. JAMA. 2017;318(16):1592-1604.

7. Yiou R, Lefaucheur JP, Atala A. The regeneration process of the striated urethral sphincter involves activation of intrinsic satellite cells. Anat Embryol (Berl). 2003;206(6):429-35.

8. Klauser A, Frauscher F, Strasser H, Helweg G, KÃ¶lle D, Strohmeyer D, Stenzl A, zur Nedden D. Age-Related Rhabdosphincter Function in Female Urinary Stress Incontinence. Journal of Ultrasound in Medicine. 2004;23(5):631-637.

9. Herrera-Imbroda B, Lara MF, Izeta A, Sievert KD, Hart ML. Stress urinary incontinence animal models as a tool to study cell-based regenerative therapies targeting the urethral sphincter. Adv Drug Deliv Rev. 2015;82-83:106-16.

10. Bennington J, Williams JK, Andersson K-E. New concepts in regenerative medicine approaches to the treatment of female stress urinary incontinence. Current Opinion in Urology. 2019;29(4):380 - 384.

11. Yiou R, Mahrouf-Yorgov M, Trebeau C, Zanaty M, Lecointe C, Souktani R, Zadigue P, Figeac F, Rodriguez AM. Delivery of human mesenchymal adipose-derived stem cells restores multiple urological dysfunctions in a rat model mimicking radical prostatectomy damages through tissue-specific paracrine mechanisms. Stem Cells. 2016;34(2):392-404.

12. Williams JK, Dean A, Badlani G, Andersson K-E. Regenerative Medicine Therapies for Stress Urinary Incontinence. The Journal of Urology. 2016;196(6):1619-1626.

13. Eberli D, Aboushwareb T, Soker S, Yoo JJ, Atala A. Muscle precursor cells for the restoration of irreversibly damaged sphincter function. Cell Transplant. 2012;21(9):2089-98.

14. Mitterberger M, Pinggera GM, Marksteiner R, Margreiter E, Plattner R, Klima G, Bartsch G, Strasser H. Functional and Histological Changes after Myoblast Injections in the Porcine Rhabdosphincter. European Urology. 2007;52(6):1736-1743.

15. Peters KM, Dmochowski RR, Carr LK, Robert M, Kaufman MR, Sirls LT, Herschorn S, Birch C, Kultgen PL, Chancellor MB. Autologous Muscle Derived Cells for Treatment of Stress Urinary Incontinence in Women. The Journal of Urology. 2014;192(2):469-476.

16. Gotoh M, Yamamoto T, Shimizu S, Matsukawa Y, Kato M, Majima T, Takai S, Funahashi Y, Toriyama K. Treatment of male stress urinary incontinence using autologous adipose-derived regenerative cells: Long-term efficacy and safety. Int J Urol. 2019;26(3):400-405.

17. Kuismanen K, Sartoneva R, Haimi S, Mannerström B, Tomás E, Miettinen S, Nieminen K. Autologous adipose stem cells in treatment of female stress urinary incontinence: results of a pilot study. Stem Cells Transl Med. 2014;3(8):936 - 941.

18. Amend B, Kelp A, Vaegler M, Klunder M, Frajs V, Klein G, Sievert KD, Sawodny O, Stenzl A, Aicher WK. Precise injection of human mesenchymal stromal cells in the urethral sphincter complex of Gottingen minipigs without unspecific bulking effects. Neurourol Urodyn. 2017;36(7):1723-1733.

19. Burdzinska A, Dybowski B, Zarychta-Wisniewska W, Kulesza A, Hawryluk J, Graczyk-Jarzynka A, Kaupa P, Gajewski Z, Paczek L. Limited accuracy of transurethral and periurethral intrasphincteric injections of cellular suspension. Neurourol Urodyn. 2018;37(5):1612-1622.

20. Jager L, Linzenbold W, Fech A, Enderle M, Abruzzese T, Stenzl A, Aicher WK. A novel waterjet technology for transurethral cystoscopic injection of viable cells in the urethral sphincter complex. Neurourol Urodyn. 2020;39(2):594-602.

21. Linzenbold W, Jager L, Stoll H, Abruzzese T, Harland N, Beziere N, Fech A, Enderle M, Amend B, Stenzl A, Aicher WK. Rapid and precise delivery of cells in the urethral sphincter complex by a novel needle-free waterjet technology. BJU Int. 2021;127(4):463-472.

22. Danalache M, Knoll J, Linzenbold W, Enderle M, Abruzzese T, Stenzl A, Aicher WK. Injection of Porcine Adipose Tissue-Derived Stromal Cells by a Novel Waterjet Technology. Int J Mol Sci. 2021;22(8).

23. Dinnyes A, Schnur A, Muenthaisong S, Bartenstein P, Burcez CT, Burton N, Cyran C, Gianello P, Kemter E, Nemeth G, Nicotra F, Prepost E, Qiu Y, Russo L, Wirth A, Wolf E, Ziegler S, Kobolak J. Integration of nano- and biotechnology for beta-cell and islet transplantation in type-1 diabetes treatment. Cell Prolif. 2020;53(5):e12785.

24. Mau M, Kalbe C, Viergutz T, Nurnberg G, Rehfeldt C. Effects of dietary isoflavones on proliferation and DNA integrity of myoblasts derived from newborn piglets. Pediatr Res. 2008;63(1):39-45.

25. Kalbe C, Mau M, Wollenhaupt K, Rehfeldt C. Evidence for estrogen receptor alpha and beta expression in skeletal muscle of pigs. Histochem Cell Biol. 2007;127(1):95-107.

26. Metzger K, Tuchscherer A, Palin MF, Ponsuksili S, Kalbe C. Establishment and validation of cell pools using primary muscle cells derived from satellite cells of pig skeletal muscle. In Vitro Cell Dev Biol Anim. 2020;56(3):193-199.

27. Kelp A, Abruzzese T, Wöhrle S, Frajs V, Aicher WK. Labeling Mesenchymal Stromal Cells with PKH26 or VybrantDil Significantly Diminishes their Migration, but does not affect their Viability, Attachment, Proliferation and Differentiation Capacities. J Tissue Sci & Engin. 2017;8:199.

28. Rasmussen R, Morrison T, Herrmann M, Wittwer C. Quantitative PCR by continuous fluorescence monitoring of a double strand dna specific binding dye. Biochemica. 1998;2:8 - 11.

29. Pfaffl MW. A new mathematical model for relative quantification in real-time RT-PCR. Nucleic Acids Res. 2001;29(9):e45.

30. Ausubel FM, Brent R, Kingston RE, Moore DD, Seidman JG, Smith JA, Struhl K. Current Protocols in Molecular Biology. In: Ausubel FM, editor. Current Protocols. New York: John Wiley & Sons; 1994.

31. Coligan JE, Kruisbeek A, Margulies DH, Shevach EM, Strober W. Current protocols in immunology. Colligan JE, editor. New York: John Wiley & Sons; 1994.

32. Linzenbold W, Fech A, Hofmann M, Aicher WK, Enderle MD. Novel Techniques to Improve Precise Cell Injection. Int J Mol Sci. 2021;22(12).

33. Kelp A, Albrecht A, Amend B, Klunder M, Rapp P, Sawodny O, Stenzl A, Aicher WK. Establishing and monitoring of urethral sphincter deficiency in a large animal model. World J Urol. 2017;35(12):1977-1986.

34. Aragon IM, Imbroda BH, Lara MaF. Cell Therapy Clinical Trials for Stress Urinary Incontinence: Current Status and Perspectives. International journal of medical sciences. 2018;15(3):195-204.

35. Barakat B, Franke K, Schakaki S, Hijazi S, Hasselhof V, Vogeli TA. Stem cell applications in regenerative medicine for stress urinary incontinence: A review of effectiveness based on clinical trials. Arab J Urol. 2020;18(3):194-205.

36. Vilsboll AW, Mouritsen JM, Jensen LP, Bodker N, Holst AW, Pennisi CP, Ehlers L. Cell-based therapy for the treatment of female stress urinary incontinence: an early cost-effectiveness analysis. Regen Med. 2018;13(3):321-330.

37. Sun X, Zhu H, Li W, Zhao L, Li W, Li X, Xie Z. Small extracellular vesicles secreted by vaginal fibroblasts exert inhibitory effect in female stress urinary incontinence through regulating the function of fibroblasts. PLoS One. 2021;16(4):e0249977.

38. Noronha NdC, Mizukami A, Caliinari-Oliveira C, Cominal JG, Rocha JLM, Covas DT, Swiech K, Malmegrim KCR. Priming approaches to improve the efficacy of mesenchymal stromal cell-based therapies. Stem Cell Research & Therapy. 2019;10(1):131.

39. Gorodetsky R, Aicher WK. Allogenic Use of Human Placenta-derived Stromal Cells as Highly Active and Competent Cells for Regenerative Therapies. Int J Mol Sci. 2021;22(10).

40. Yiou R, Dreyfus P, Chopin DK, Abbou CC, Lefaucheur JP. Muscle precursor cell autografting in a murine model of urethral sphincter injury. BJU Int. 2002;89(3):298-302.

41. Gräs S, Klarskov N, Lose G. Intraurethral injection of autologous minced skeletal muscle: a simple surgical treatment for stress urinary incontinence. J Urol. 2014;192(3):850-5.

42. Yokoyama T, Yoshimura N, Dhir R, Qu Z, Fraser MO, Kumon H, de Groat WC, Huard J, Chancellor MB. Persistence and survival of autologous muscle derived cells versus bovine collagen as potential treatment of stress urinary incontinence. J Urol. 2001;165(1):271-6.

43. Yasuda T, Weisel RD, Kiani C, Mickle DA, Maganti M, Li RK. Quantitative analysis of survival of transplanted smooth muscle cells with real-time polymerase chain reaction. J Thorac Cardiovasc Surg. 2005;129(4):904-11.

44. Marquardt LM, Heilshorn SC. Design of Injectable Materials to Improve Stem Cell Transplantation. Current Stem Cell Reports. 2016;2(3):207-220.

45. Habib M, Shapira-Schweitzer K, Caspi O, Gepstein A, Arbel G, Aronson D, Seliktar D, Gepstein L. A combined cell therapy and in-situ tissue-engineering approach for myocardial repair. Biomaterials. 2011;32:7514-23.

46. Hashemzadeh MR, Taghavizadeh Yazdi ME, Amiri MS, Mousavi SH. Stem cell therapy in the heart: Biomaterials as a key route. Tissue and Cell. 2021;71:101504.

47. Luan C, Liu P, Chen R, Chen B. Hydrogel based 3D carriers in the application of stem cell therapy by direct injection. Nanotechnology Reviews. 2017;6(5):435-448.

48. Jankowski RJ, Deasy BM, Huard J. Muscle-derived stem cells. Gene Ther. 2002;9(10):642-7.

49. Koraitim MM. The Male Urethral Sphincter Complex Revisited: An Anatomical Concept and its Physiological Correlate. Journal of Urology. 2008;179(5):1683-1689.

50. Schreiber J. Investigation of the penetration depth of microparticles by water jet application in porcine urethra [German]. Tübingen: Eberhard -Karls-Universität; 2020.

51. Beck F. Penetration depth and distribution of nanoparticles after injection into the porcine urethra using a new water jet technology [German]. Tübingen: Eberhard-Karls-Universität; 2020.

52. Enosawa S, Kobayashi E. Controllable Immunosuppression in Pigs as a Basis for Preclinical Studies on Human Cell Therapy. In: Miyagawa S, editor. Xenotransplantaton - Comprehensive Study. Volume 1. Osaka: Osaka Univesity; 2020.

53. Castro R, Libanio D, Pita I, Dinis-Ribeiro M. Solutions for submucosal injection: What to choose and how to do it. World J Gastroenterol. 2019;25(7):777-788.

54. Jacques J, Kerever S, Carrier P, Couquet CY, Debette-Gratien M, Tabouret T, Lepetit H, Geyl S, Loustaud-Ratti V, Sautereau D, Legros R. HybridKnife high-pressure glycerol jet injection for endoscopic submucosal dissection increases procedural ease and speed: a randomised study in pigs and a human case series. Surg Endosc. 2016;30(7):3152-9.

55. Repici A, Maselli R, Carrara S, Anderloni A, Enderle M, Hassan C. Standard needle versus needleless injection modality: animal study on different fluids for submucosal elevation. Gastrointest Endosc. 2017;86(3):553-558.

56. Amer MH, White LJ, Shakesheff KM. The effect of injection using narrow-bore needles on mammalian cells: administration and formulation considerations for cell therapies. J Pharm Pharmacol. 2015;67(5):640-50.

57. FELASA. <https://felasa.eu/working-groups/guidelines>.

58. Maak S, Wicke M. Analysis of gene expression in specific muscles of swine and turkey. Arch. Tierz. 2005;48:135 - 140.

59. Kalbe C, Mau M, Rehfeldt C. Developmental changes and the impact of isoflavones on mRNA expression of IGF-I receptor, EGF receptor and related growth factors in porcine skeletal muscle cell cultures. Growth Horm IGF Res. 2008;18(5):424-33.

60. Jaillard S, Holder-Espinasse M, Hubert T, Copin MC, Duterque-Coquillaud M, Wurtz A, Marquette CH. Tracheal replacement by allogenic aorta in the pig. Chest. 2006;130(5):1397-404.

**Legends to the figures**

**Figure 1*:*** *Schematic Overview on Cell Injections.*

For cell injections in cadaveric urethra samples, the urethra was opened, placed on a sponge with the epithelium (i.e., urothelial cell layer) facing up. **(A)** Injections in the submucosal layer by Williams needle (WN) were performed at an angle of approx. 30° to 45°. For cell injections, the tip of the WN was inserted in the tissue for a few millimeters. Injections by WJ were performed vertical. The tip of the WJ lance was lowered by a gauge to the surface of the urothelial layer and moved 2 mm down w/o tissue penetration to avoid loss of cells by splash to the side caused by the Bernoulli effect. The X- and Y-dimensions for histologic evaluation are explained in the inserts on top. **(B)** For transurethral cell injections in living animals by aid of cystoscope under visual control, WJ injections were performed. After slightly tilting the device in the urethra, the flexible tip of the injection lance enabled angulated WJ injections in the urethra w/o penetration of the urothelium. **(C)** Schematic overview for determination of the distribution of cells in the tissue targeted and determination distance between sphincter muscle and injected cells (DISIC).

**Figure 2:** Characterization of Porcine Muscle-derived Cells

**(A)** Muscle derived cells (MDCs) were isolated and expanded *in vitro.* The proliferating MDCs appeared as adherent populations as exemplified for the first three passages P1 to P3. Size bars = 100 μm. **(B)** Expression of myogenic marker transcripts was explored by RT-qPCR to compare the populations isolated from young wildtype (WT) and transgenic (TG) piglets. The graphic presents the normalized mean transcripts amounts and standard deviations (Y-axis) of 7 genes as indicated (X-axis; WT: n=1, TG: n=4). **(C)** Expression of myogenic marker proteins desmin, fast twitch and slow twitch myosin in WT and TG MDCs with specific primary antibodies, followed by counterstaining with FITC- or Alexa 488- labelled detection antibodies (green). Nuclei were counterstained by DAPI (blue). Both populations expressed desmin. Fast myosin was expressed by WT MDCs at low and by TG MDCs at moderate levels. Slow myosin was detected only on a few cells from TG piglets. Size bars = 50 μm. Cells reacted with detection antibodies only served as controls (c).

**Figure 3:** Cell Injections in Cadaveric Tissue Samples

**(A)** Examples of calcein-AM-labelled MDCs injected by waterjet (WJ) or Williams needle (WN) in fresh porcine cadaveric urethra samples, aspirated, washed and seeded in 6-well plates for further expansion in culture. Significant differences between yield of viable cells and their proliferation rates were not observed. Not injected calcein-AM-labelled MDCs served as controls. The pictures are representative artwork from a 5 day follow-up. Size bars indicate 50 μm. **(B)** TG MDCs were injected by WN in fresh porcine cadaveric urethra samples. Cryosections were generated and stained by fluorescent phalloidin to visualize the injected cells and muscular tissue. The sphincter muscle and injected MDCs appear green in the stitched overview (10x objective, left, size bar 2 mm). Injected TG MDCs appear yellow in the magnified micrograph (20x objective, right, size bar 0.2 mm). **(C)** TG MDCs were injected by WJ in fresh porcine cadaveric urethra samples. Cryosections were generated and stained by fluorescent phalloidin to visualize the injected cells and muscular tissue. The sphincter muscle and injected MDCs appear green in the stitched overview (10x objective, left, size bar 2 mm). Injected TG MDCs appear yellow in the magnified micrograph (20x objective, right, size bar 0.5 mm). Cell nuclei were counterstained by DAPI (blue).

**Figure 4:** Waterjet Injections in Living Animals at Moderate Pressure Levels

Stitched micrographs (10x objective) of complete cryosection samples document PKH26-labelled fluorescent red cells in pigs treated by WJ and E60-10 protocol **(A)** prepared on day 1, **(B)** on day 3, and **(C)** on day 7 after injections. Cells are localized in the submucosa. Muscle tissue is stained by phalloidin and appears green, cell nuclei are counterstained by DAPI and appear blue. Size bars = 2 mm. **(D)** By larger magnification (40x objective) PKH26 fluorescent cell somata surrounded by defined nuclei and detection of expression of recombinant GFP suggest that cells injected are intact and alive after 3 days of follow-up. **(E)** PKH26 labelled cells were also detected after 7 days of follow-up. Size bars = 20 μm.

**Figure 5:** Waterjet Injections in Living Animals with Elevated Pressure Levels

Stitched micrographs (10x objective, size bar = 2 mm) of complete cryosection samples document PKH26-labelled fluorescent red cells in pigs treated by WJ and E80-10 protocol **(A)** on day 3 after injection. Cells are localized in the submucosa closer to the muscle. Muscle tissue is stained by phalloidin and appears green, cell nuclei are counterstained by DAPI and appear blue. **(B)** By larger magnification (40x objective, size bar = 20 μm) PKH26 labelled fluorescent cell somata surrounded by defined nuclei indicate that injected cells are intact.

**Figure 6:** Three-dimensional Cell Distribution in Tissues Targeted

**(A)** Consecutive stacks of cryosections were investigated to determine the distribution of cells in 3 dimensions after injection by WJ using the E60-10 or E80-10 protocol in living animals (LA) and after injection in cadaveric tissue samples (CS) by WJ using the E60-10 protocol or by WN as indicated. In the X- and Y-axis, only minor differences were measured. Distribution of MDCs in the height (i.e., Z-axis) was significantly higher after WJ E80-10 injections in living animals when compared to WJ E60-10 injections. WJ E60-10 injections in living animals yielded a significantly higher cell distribution when compared to WJ E60-10 injections of MDCs in cadaveric tissue samples. Due to the angular WN injection in cadaveric samples, MDC distribution along the X-axis was significantly higher after WN injection in cadaver tissue samples when compared to perpendicular WJ E60-10 injections. **(B)** Distribution of cells was also investigated in XY-, YZ-, and XZ-planes as indicated. WJ E80-10 injections in living animals showed a significantly wider distribution in the YZ-plane when compared to WJ 60-10 injections. Comparably in the XZ-plane, WJ E80-10 injections showed a significantly wider distribution when compared to WJ 60-10 injections. WJ E60-10 LA injections yielded significantly larger distribution in the XZ-plane when compared to injections in cadaveric urethra samples. **(C)** The distance between the sphincter muscle and the injected cells (DISIC) was significantly higher after WJ E60-10 injections in living animals when compared to WJ E80-10 injections.

**Figure 7:** Injection Depth Reached by Waterjet in Living Animals

**(A)** Cryosamples were generated after WJ injections in living animals and stained by phalloidin and DAPI to label muscle in green and cell nuclei in blue. WJ E80-10 injection of MDCs caused in 1/6 pigs full penetrations on both lateral injection spots. In one penetration spot, PKH26 labelled MDCs were not detected (yellow arrow), while on the other site PKH26 labelled MDCs were observed outside the urethra (white arrow). **(B)** WJ E60-10 injections yielded in 1/18 pig a deep penetration and PKH26-labelled MDCs were detected in the muscular layers of the sphincter complex. Size bars = 2 mm.

**Figure 8:** Histological Analysis of the Urethral Tissue after Waterjet Injection

**(A)** Cryosections from layers containing WJ injected cells from animals after 7 days of follow-up were stained by AZAN to detect the injected MDCs (red color) within the submucosal connective tissue (blue color; stitched overview; 2.5x objective, size bar = 2 mm). The area of the magnified picture on the left is marked by rectangle. **(B)** Magnified aspect of the area of MDC injection as indicated (20x objective, size bar 50 μm). **(C)** Cryosections from layers containing WJ injected cells from animals after 7 days of follow-up were stained by HE to visualize the tissue structure and to detect infiltration of inflammatory cells (stitched overview; 2.5x objective, size bar 2 mm). The area of the magnified picture on the left is marked by rectangle. **(D)** Magnified aspect of the area of MDC injection as indicated does not show infiltration of mononuclear cells (20x objective, size bar = 50 μm).

**Figure 9:** Amplification of the Male-specific *SRY-*gene by PCR from Cryosections

DNA was prepared to detect the intact *SRY* gene by PCR after WJ of MDCs using the **(A)** E60-10 method at day 1 of f/u, **(B)** E60-10 method on day 3 of f/u, **(C)** E60-10 method on day 7 of f/u, **(D)** E80-10 method on day 3 of f/u. “M” denotes the 100 bp DNA ladder size marker; “**>”** = 600 bp, “**_*_“** = 200 bp, “**+**” = positive control (DNA from porcine male adipocytes), “-“ = negative control (DNA from female porcine adipocytes); “➝” the 133 bp *SRY* PCR product. DNA samples from animals are numbered. Note that for E60-10 at 3 days f/u, cells were found only in 5/6 animals treated.

**Tables:**

**Table 1:**

| **Gene** | **Upper Primer** | **Lower Primer** | **Size** | **Acc.. Number** | **Ref.** |
| --- | --- | --- | --- | --- | --- |
| *ACTA2* | CGGGCAGGTCATCACCATC | CGTGTTGGCGTAGAGGTCCTT | 160 | [NM_001164650.1](https://www.ncbi.nlm.nih.gov/entrez/viewer.fcgi?db=nucleotide&id=257470978) | ^58^ |
| *DES* | ACACCTCAAGGATGAGATGGC | CAGGGCTTGTTTCTCGGAAG | 176 | [NM_001001535.1](https://www.ncbi.nlm.nih.gov/entrez/viewer.fcgi?db=nucleotide&id=48374062) |  |
| *MYF5* | GCTGCTGAGGGAACAGGTGGA | CTGCTGTTCTTTCGGGACCAGAC | 135 | [NM_001278775.1](https://www.ncbi.nlm.nih.gov/entrez/viewer.fcgi?db=nucleotide&id=523580059) | ^58^ |
| *MYF6* | CGCCATCAACTACATCGAGAGGT | ATCACGAGCCCCCTGGAAT | 189 | [NM_001244672.1](https://www.ncbi.nlm.nih.gov/entrez/viewer.fcgi?db=nucleotide&id=347658935) | ^58^ |
| *MYL1* | CTCTCAAGATCAAGCACTGCG | GCAGACACTTGGTTTGTGTGG | 198 | [NM_214374.2](https://www.ncbi.nlm.nih.gov/entrez/viewer.fcgi?db=nucleotide&id=157427686) | ^58^ |
| *MYOD1* | CACTACAGCGGTGACTCAGACGCA | GACCGGGGTCGCTGGGCGCCTCGCT | 145 | [NM_001002824.1](https://www.ncbi.nlm.nih.gov/entrez/viewer.fcgi?db=nucleotide&id=50657387) | ^58^ |
| *MSTN* | CCCGTCAAGACTCCTACAACA | CACATCAATGCTCTGCCAA | 141 | [NM_214435.2](https://www.ncbi.nlm.nih.gov/entrez/viewer.fcgi?db=nucleotide&id=51783958) | ^58^ |
| *B2M* | ACGGAAAGCCAAATTACCTGAACTG | TCTGTGATGCCGGTTAGTGGTCT | 261 | [NM_213978.1](https://www.ncbi.nlm.nih.gov/entrez/viewer.fcgi?db=nucleotide&id=47522781) | ^59^ |
| *SRY* | GACAATCATAGCTCAAACGATG | TCTCTAGAGCCACTTTTCTCC | 133 | [NC_010462.3](https://www.ncbi.nlm.nih.gov/nucleotide/1154346151?from=40485451&to=40554255&report=gbwithparts) | ^60^ |

**PCR primer pairs for specific amplification of porcine cDNA and chromosomal DNA.** The column “Gene” refers chromosomal DNA or cDNA, al primers in 5’>3’ orientation, column “Size” refers to PCR product lengths according to the published sequences in base pairs (bp) and confirmed by electrophoresis in agarose gels, column “Accession Number” denotes the gene bank accession numbers ([www.ncbi.nlm.nih.gov](http://www.ncbi.nlm.nih.gov)), and Ref. a citation where applicable.

**Table 2:**

| **Antigen** | **Antibody** | **Source** | **Label** | **Dilution** | **Company** |
| --- | --- | --- | --- | --- | --- |
| Desmin | IgG, pAB | Rabbit | ø | 1:200 | Abcam 15200 |
| Fast myosin | Serum | Rabbit | ø | 1:200 | Abcam 91506 |
| Slow myosin | IgG, mAB | Mouse | ø | 1:100 | Abcam 11083 |
| Rabbit IgG | IgG, pAB F(ab’)_2_ | Donkey | FITC | 1:100 | Jackson 711-096-152 |
| Mouse IgG | IgG, pAB F(ab’)_2_ | Donkey | Alexa Fluor 488 | 1:2000 | Abcam181289 |

**Antibodies for immunocytochemistry and immunohistochemistry.** Antibodies utilized as primary antibodies (lines 1 – 3) and for detection of primary antibodies (lines 4 ,4) of cells in chamber slides or on cryosection. pAB: polyclonal antibody, mAB: monoclonal antibody

# Supplementary Material

Supplementary figures S1, S2, S3 and the corresponding legends.

**Legends to the figures in the supplement**

**Figure S1:** Determination of Urothelial Muscle Strength by Urodynamics

The urethral muscle strength and closure function was determined by a sensor and urodynamics. The scale on this sensor facilitated the injection of MDCs by WJ in the area of maximal muscle strength. The maximal urethral closure pressure (p_max_) describes the location of main sphincter muscle in the urethra. The functional urethral length describes the part in which the closure pressure is higher than the intravesical pressure. The area under the curve (AUC) describes the integral of urethral wall pressures over the functional length. The arrow points at the WJ injection site.

**Figure S2:** Pressure Profiles of the Waterjet System

An upgraded pump and controller system (UPaCS) was operated in an improved pressure control mode (IPCM) to facilitate a short water impulse at elevated pressures ranging from E40 to E80, which is an equivalent of approx. 40 to 80 bars, respectively (= phase I). By this water impulse the tissue targeted is stretched and microchannels are opened in phase I. Within milliseconds pressure levels are reduced to E5 – E10 (= phase II). This low pressure is optimal for injection of viable cells in the open microchannels with high efficacy in phase II. In this study, pressure profiles of E80-10 (dashed line) and E60-10 (solid line) were employed.

**Figure S3:** Localization of Injected MDCs in the Urethra by IVIS Imaging

Fluorescent MDCs were localized in the urethrae after *in vivo* injections by an In Vivo Imaging System (IVIS) and visualized by a false-color heat map, while the remaining organ is shown in grey. This facilitated fast preparations of frozen tissue samples from the region of interest and further processing of the samples.
