# Supplementary material for: Replacing Needle Injection by a Novel Waterjet Technology Grants Improved Muscle Cell Delivery in Target Tissues
Source: Cell Transplant. 2022 Apr 23;31:09636897221080943. doi: 10.1177/09636897221080943 (PMC9036380; doi:10.1177/09636897221080943)
Supplement: sj-docx-1-cll-10.1177_09636897221080943 – Supplemental material for Replacing Needle Injection by a Novel Waterjet Technology Grants Improved Muscle Cell Delivery in Target Tissues [file sj-docx-1-cll-10.1177_09636897221080943.docx]

Tübingen, 8.11. 2021

To the

Editorial Board

Cell Transplantation

To whom it might concern:

Dear Madam /Sir,

On behalf of all authors, I would like to thank the Editorial Board and the reviewers of the journal *Cell Transplantation* that our manuscript entitled

**Replacing needle injection by a novel waterjet technology**

**grants improved muscle cell delivery in target tissues**

was reviewed. We appreciate the comments we received to the original version and revised the manuscript accordingly (see below). We hope that our manuscript now merits publication in *Cell Transplantation.*

Attached please the revised version of the manuscript generated by “follow-changes-mode” (i.e., Geng-main-R1-changes). The changes are marked in red letters. As requested, we also include a clean version of the revised document (i.e., Geng-main-R1 clean). The artwork was not revised.

If the reviewers of the Editorial Board needed some additional documents to proceed with the review process, feel free to contact me at your convenience.

On behalf of all authors,

W. Aicher

Appendix:

Comments to the Author & Reply

Critique and reply:

Critique reviewer 1: This study describes the development and in vitro and in vivo (using pig model) characterization of a novel cell injection technology to deliver viable allogeneic cells in the urethral sphincter complex by waterjet delivery. Newly generated data on waterjet cell delivery were also compared to a standard injection needle-coupled cell delivery approach. Overall this new cell-delivery technology is associated with a significantly wider delivered-cell distribution when compared to needle injections with the success rates of waterjet cell application in living animals being highly significant compared to needle injections. As such this novel technique appears to provide a significant advantage over currently used needle cell delivery.
I have only one minor point.

It is likely that a multiple delivery regions will be targeted in clinic and that an increased number of delivered cells can accelerate and potentiate the expected treatment effect. A comment on how many potential waterjet-delivered cell deposits can be performed in one surgical session would be helpful to understand a potential clinical use of this delivery approach.

Our reply:

We thank the reviewer for the critiques and suggestions. We fully agree with these comments and revised the manuscript in last paragraph of the discussion on page 17 as follows (changes in blue letters):

To examine the efficacy of the WJ technology for the clinical use intended, future experiments must include additional studies to determine optimal cell doses, single versus multiple injections in one session, repeating cell injections during follow-up, and possibly other therapies in a suitable animal model documenting functional sphincter regeneration. To this end, a porcine model of urinary incontinence was developed recently^33^. But these aspects were not yet addressed in our current studies.
